# Operant conditioning in Indian free-ranging dogs: effects of positive and threatening cues on sociability

**DOI:** 10.1098/rsos.261465

**Published:** 2026-11-11

**Authors:** Srijaya Nandi, Dipanjan Roy, Abantika Adak, Rohan Sarkar, Anindita Bhadra

**Affiliations:** ^1^ Department of Biological Sciences, Indian Institute of Science Education and Research, Kolkata, West Bengal, India; ^2^ Department of Biotechnology, Maulana Abul Kalam Azad University of Technology, Kolkata, West Bengal, India

**Keywords:** operant conditioning, dog–human interactions, behavioural plasticity, sociability, free-ranging dogs

## Abstract

Sociability towards humans is an adaptive trait in free-ranging dogs (FRDs), enabling them to access resources while navigating the risky anthropogenic habitat. We investigated whether operant conditioning shapes sociability in Indian FRDs and whether learned responses generalize to an unfamiliar individual. We exposed 54 dog groups to either a positive or a threatening cue over five consecutive days, followed by tests on day 6 with an unfamiliar and then the familiar experimenter. Using Bayesian GLMMs, we analysed approach proportion, approach latency and demeanour. The threatening cue reduced approach behaviour, increased approach latency and caused a shift towards neutral responses to the familiar experimenter, with the changes occurring earlier compared with the positive cue. The positive cue reduced approach latency towards the familiar experimenter, but did not increase the proportion of approach. Towards the unfamiliar experimenter on day 6, positive-cue groups approached more often, threatening-cue groups approached more slowly than on day 1, and demeanour changes under the threatening cue were specific to the familiar experimenter. Our results suggest that FRDs adjust their behaviour primarily towards the person associated with an outcome, which may help them balance resource acquisition with risk avoidance. These insights have implications for informing management strategies to enable dog–human coexistence.

## 1. Introduction

Sociability is a core personality trait in animals [1], defined as the tendency of an individual to associate with others in contexts not directly related to reproduction or aggression [2]. Such associations can occur both within and across species and may serve a range of adaptive functions, including reducing predation risk, facilitating ectoparasite removal, improving foraging success and conserving energy [3]. For animals coexisting with humans, as in the case of urbanized and domesticated animals, sociability towards people can be particularly beneficial, as it often improves access to resources. However, approaching unfamiliar humans carries inherent risks, and poor decision-making in this regard can lead to severe negative outcomes.

One mechanism that may shape sociability in these contexts is operant conditioning, a form of learning in which the future likelihood of a behaviour depends on the immediate consequence it leads to [4]. Behaviours followed by reinforcement tend to increase in frequency, whereas those followed by punishment typically decrease [5]. Operant conditioning has been documented across a broad range of taxa, from invertebrates [6] to vertebrates [7]. It plays a major role in the development of behavioural plasticity among animals, along with other factors such as evolution and cultural transmission [8].

Learning by operant conditioning has been studied in several domesticated species such as pet dogs [9], goats [10], cows [11] and horses [12]. Free-ranging dogs (FRDs) represent a particularly interesting population for studying how human-directed sociability is shaped by operant conditioning. Because FRDs are not directly supervised, their daily movements and activities occur independently of human control [13]. Nonetheless, they receive both positive and negative interactions from the humans they encounter, and these interactions shape their behavioural responses [14]. They have coexisted alongside humans for centuries across all possible human habitats [15] and account for roughly 80% of the global dog population [16,17]. They are largely dependent on human-generated waste for their sustenance and also tend to give birth near human habitations [18]. Previous research has shown that Indian FRDs increase their sociability towards humans over repeated interactions [19,20], and adult FRDs respond differently in pointing gesture following tasks when they are petted by a human compared with when not [21]. However, such studies focused on dogs that initially approached an unfamiliar experimenter, leaving open the question of whether sociability can also be modified in non-approachable individuals.

In the present study, we investigated whether operant conditioning contributes to the sociability of Indian FRDs towards unfamiliar humans. Specifically, we examined two contingencies that they commonly encounter in their daily environment: receiving food (positive cue) and exposure to an aversive stimulus such as a stick (threatening cue). We also systematically measured changes in their sociability over repeated trials with both a familiar and an unfamiliar experimenter. We further aimed to assess whether these forms of conditioning differ in how quickly their effects emerge, thereby providing insight into the learning dynamics underlying dog–human interactions. This comparison is critical, as organisms often exhibit a ‘negativity bias,’ where learning to avoid a threat occurs more rapidly and robustly than learning to approach a reward, given the higher potential cost of a negative outcome [22]. Finally, we asked whether changes in sociability generalize across individuals, or whether they remain specific to the person involved in repeated interactions. Our study therefore addressed four key questions. (i) Does a threatening cue reduce sociability over repeated interactions with the familiar experimenter? (ii) Does a positive cue enhance sociability over repeated interactions with the familiar experimenter? (iii) Does the change in sociability emerge earlier under the threatening cue than under the positive cue during repeated interactions with the familiar experimenter? (iv) Do these effects extend to an unfamiliar experimenter or remain tied only to the familiar experimenter?

Based on the principles of operant conditioning, we formulated four primary hypotheses. First, we predicted that dogs in the threatening cue group would show a decrease in sociability towards the familiar experimenter, as the aversive stimulus would discourage approach behaviour. Second, we hypothesized that dogs in the positive cue group would display an increase in sociability, as they learn to associate the experimenter with a food reward. Third, based on the principle of negativity bias, we hypothesized that behavioural changes would emerge earlier under the threatening cue than under the positive cue. Finally, we predicted that these learned responses would not generalize beyond the familiar experimenter. Because FRDs have been reported to learn to discriminate between a rewarding and a non-rewarding human after four brief interactions [20], we expected dogs to distinguish the person associated with the consequence from the unfamiliar experimenter, and therefore did not expect sociability towards the unfamiliar experimenter to change.

## 2. Methodology

### 2.1. Subjects

We selected 54 free-ranging dog groups for the study. Groups were defined as three or more dogs located within approximately five meters of each other on the first day of the experiment. Inclusion criteria required a minimum of three visibly healthy adults per group. A male was classified as an adult if his testes were descended, and a female was classified as an adult if her nipples were darkened. All dogs present in a group were recorded; one group included one pup and two juveniles, which were retained in the analyses. In total, 27 groups were randomly assigned to receive a positive cue and 27 groups to receive a threatening cue. These groups comprised 289 individually identified dogs (152 in positive-cue groups and 137 in threatening-cue groups; 130 females and 159 males). All dogs were photographed for later identification, and group locations were recorded using a GPS-enabled mobile phone. Adjacent groups were not tested, as their behaviour might have been influenced by observing earlier trials on neighbouring groups.

### 2.2. Study areas

Experiments were conducted in four urban and semi-urban sites in West Bengal, India: Gayeshpur (22°57′29″ N, 88°29′33″ E), Anandanagar (22°58′44″N, 88°29′15″E), Kataganj (22°56′58″ N, 88°28′14″ E), and Kanchrapara (22°56′35.13″ N, 88°26′10.01″ E). A map showing the study areas is provided in figure S1 in the supplementary material.

### 2.3. Experimenters involved

Three experimenters, all unfamiliar with the focal dog groups, were involved. Experimenter 1 (E1, female) conducted the experiments from days 1–6. Experimenter 2 (E2, female) was involved only on day 6 and acted as the unfamiliar person. Experimenter 3 (E3, male) was responsible for video recording throughout the study.

### 2.4. Experimental procedure

#### 2.4.1. Approachability test (days 1–5)

On day 1, E1 initiated an approachability test by standing approximately 1.5 m from the nearest dog in a group. She produced a standardized positive vocalization, ‘ae-ae-ae’ [19] for 5 seconds to attract the attention of the dogs, and then maintained eye contact with the dogs for 25 seconds (supplementary material, video S1). Each group then received either a positive or a threatening cue, depending on its assignment. The same protocol was repeated from day 2 to 5.

**Positive cue:** following the approachability test, E1 placed Parle-G (glucose) biscuits near the dogs that approached (supplementary material, video S2). An approach was considered when a dog came within one dog body length (approx. 0.8 m) of the experimenter. The number of biscuits matched the number of dogs that had approached. She then threw biscuits towards the non-approaching dogs from a distance, ensuring that the dogs had to approach the biscuits to interact with them. Dogs were allowed up to 30 seconds to respond to the biscuits, which included behaviours like sniffing, licking or eating.

**Threatening cue:** following the approachability test, E1 displayed a 45 cm wooden stick for 30 seconds to the dogs that had approached (supplementary material, video S3). Dogs that did not approach could also observe the stick from a distance. The stick was kept concealed at the back of the experimenter's trousers and drawn out suddenly in a jerky motion and held up, similar to the way people on the streets display sticks to the dogs to threaten them.

#### 2.4.2. Approachability test by an unfamiliar person (day 6)

On day 6, E2 (unfamiliar to the dog groups) conducted the approachability test using the same procedure as E1. Immediately afterwards, she left the site and remained hidden. After a 5-minute interval, E1 repeated the test with the same group. This design allowed us to examine whether changes in sociability can be generalized to an unfamiliar individual or remain specific to the familiar experimenter.

A neutral face was maintained by the experimenters both during the approachability test and the test phase. The approachability tests conducted on day 6 by E2 and E1 were labelled 6A and 6B respectively. No cue was given after the day 6A and day 6B approachability tests. E1 did not carry any food with her for the groups that were administered the threatening cue. The experimental protocol is illustrated in figure 1.

**Figure 1.**
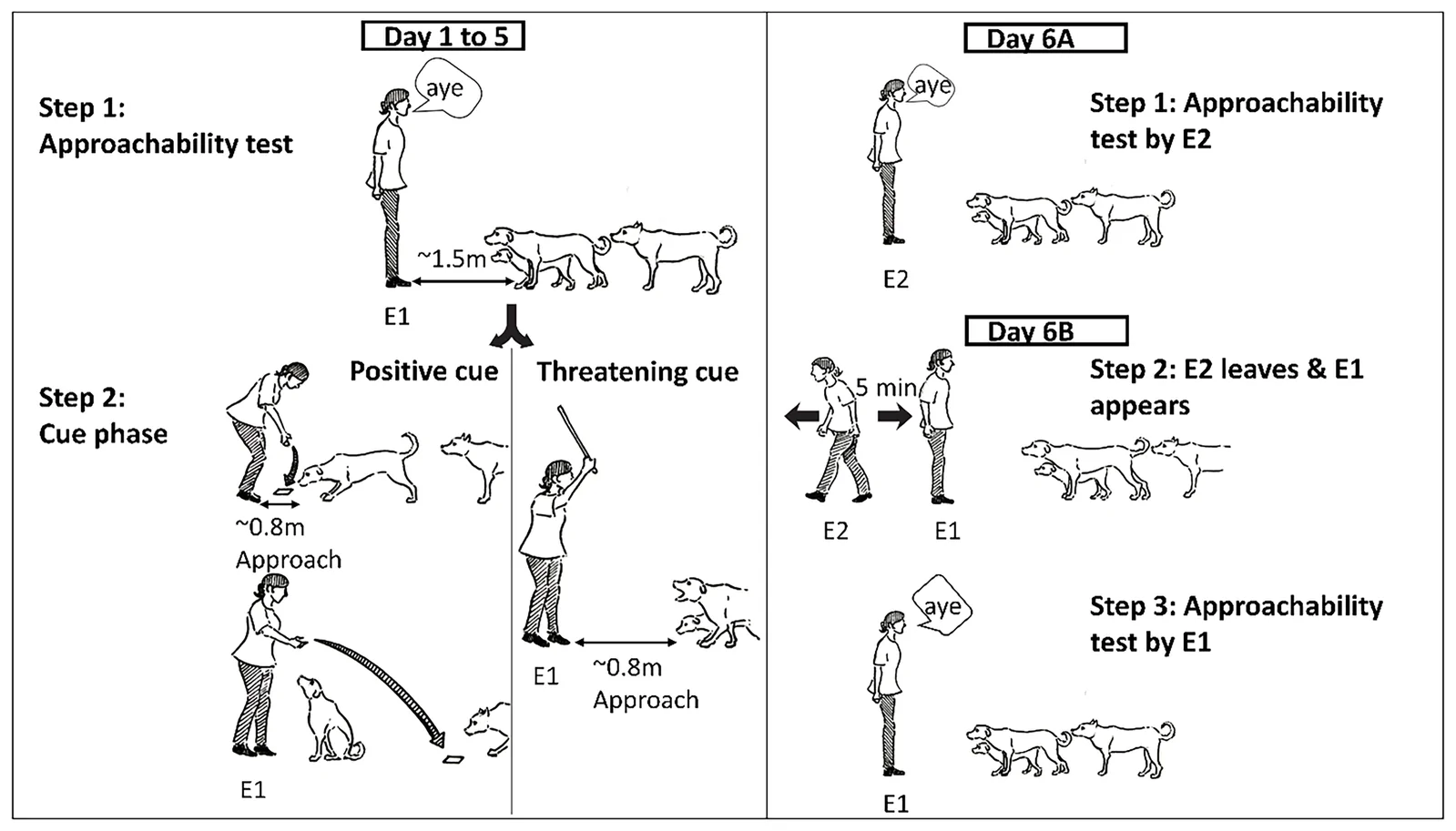
Schematic representation of the experimental protocol. Illustration by Arpan Bhattacharyya.

### 2.5. Data coding

The following behavioural parameters were coded from video recordings.

**Proportion of approach (approachability test):** the proportion of approach was calculated as the number of dogs that approached divided by the total number of dogs present at the start of the test.

**Approach latency (approachability test):** approach latency was defined as the time (in seconds) taken by a dog to approach within one dog body length (approx. 0.8 m) of the experimenter from the moment the experimenter first called the dogs. Dogs that did not approach within the allotted time were treated as censored, and their approach latency was recorded as the maximum allowed duration to approach (30 seconds).

**Demeanour (approachability and test phases):** following Bhattacharjee *et al.* [14], demeanour was classified into four categories: affiliative (e.g. tail wagging), anxious (e.g. tail tucked or lowered), aggressive (e.g. barking, growling), and neutral (behaviours that were neither affiliative, anxious nor aggressive). Only the initial demeanour displayed during each phase was considered.

All videos were coded by D.R. A.A. independently coded 20% of the videos selected at random to assess inter-rater reliability. Agreement between observers was high for both approach behaviour (Cohen's *κ* = 0.87) and demeanour classification (Cohen's *κ* = 0.85).

### 2.6. Statistical analysis

All the analyses were conducted in a Bayesian framework using generalized linear mixed-effects models (GLMMs) fitted with the brms package [23] in R version 4.2.3 [24]. For all the models, we used weakly informative priors: a normal distribution (mean = 0, s.d. = 1) on the fixed effects and an exponential distribution (rate = 1) on the standard deviations of the random effects. Each model was run with four Markov chains of 5000 iterations, the first 1000 of which were discarded as warmup, yielding 16 000 post-warmup posterior draws per model (adapt_delta = 0.95). We assessed convergence by visually inspecting trace plots and autocorrelation plots, and by confirming that all parameters had R-hat ≤ 1.01. Sampling efficiency was assessed using bulk and tail effective sample sizes (ESS), and no divergent transitions occurred. We evaluated model fit with posterior predictive checks, comparing 100 simulated datasets with the observed data. For the approach-latency model, we compared exponential, Weibull, lognormal and gamma error distributions using leave-one-out cross-validation (LOO) (loo package [25]) and retained the lognormal model, which had the best predictive performance. Differences between factor levels were assessed as differences in predicted proportions (zero-one inflated beta model), log latency (lognormal model) and category probabilities (multinomial models). For the zero-one inflated beta and lognormal models, we calculated pairwise contrasts with the emmeans package [26] using all posterior draws. Models were fitted with a fixed sampler seed. For the multinomial models, we calculated contrasts from 2000 randomly selected posterior draws of the predicted probabilities, obtained with the tidybayes package [27], using a fixed random seed to ensure reproducibility. We summarized all contrasts as posterior medians with 95% highest posterior density (HPD) intervals, computed with emmeans for the beta and lognormal models and with the bayestestR package [28] for the multinomial models, and treated an HPD interval that did not overlap zero as evidence of a difference between levels.

Pairwise comparisons were restricted to contrasts of primary interest: day 1 versus all subsequent days (days 2–5, 6A and 6B), and a comparison between day 6A and day 6B.

The four models were as follows.

**Model 1: zero-one inflated beta regression model examining factors affecting approach proportion during the approachability test.**

The proportion of dogs that approached during the approachability test (approach_proportion) was analysed using a zero-one inflated beta regression model. The fixed effects included day (seven levels: day 1, day 2, day 3, day 4, day 5, day 6a, and day 6B), cue (two levels: positive and threatening) and their interaction (day × cue). A random intercept for group identity (group_id) was included to account for repeated observations within groups. Pairwise comparisons were reported as median differences in the expected proportion of approach with 95% HPD intervals.

The model structure was:

$$\begin{array}{r} approach\_proportion∼day\times cue+\left(1|group\_id\right). \end{array}$$

**Model 2: lognormal survival model examining factors affecting approach latency during the approachability test.**

The latency to approach (time_pv) was analysed using a lognormal survival model, accounting for right-censoring via the ‘cens_brms’ indicator. The fixed effects included day, cue, sex and the interaction between day and cue. To account for the hierarchical structure of the data, random intercepts were included for group identity (group_id) and individual dogs nested within groups (group_dog). Pairwise comparisons were reported as median differences in expected log approach latency with 95% HPD intervals.

The model structure was:

$$\begin{array}{r} time\_pv|cens\left(cens\_brms\right)∼day\times \;cue+sex+\left(1|group\_id/group\_dog\right). \end{array}$$

**Model 3: multinomial regression model examining factors affecting demeanour during the approachability test and model 4: multinomial regression model examining factors affecting demeanour during the test phase.**

Demeanour, a categorical response variable, was analysed using a multinomial regression model. The fixed effects included cue, day, sex and the interaction between cue and day.

Model 3 included seven levels for day (day 1, day 2, day 3, day 4, day 5, day 6A and day 6B), while model 4 included five levels for day (day 1, day 2, day 3, day 4 and day 5).

To account for the hierarchical structure of the data, random intercepts were included for group identity (group_id) and individual dogs nested within groups (group_dog). Estimated marginal contrasts of predicted probabilities were reported as median differences in predicted probability between conditions, with 95% HPD intervals used to quantify uncertainty.

The model structure was:

$$\begin{array}{r} demeanour∼cue\times day+sex+\left(1|group\_id/group\_dog\right). \end{array}$$

## 3. Results

### 3.1. What factors affected the proportion of approach during the approachability test?

The regression model revealed a strong interaction between cue type and test day in shaping approach behaviour (table 1).

**Table 1.** Posterior parameter estimates (posterior mean (estimate), posterior standard deviation (est.Error) and 95% credible intervals) from the zero-one-inflated beta model assessing the effects of day, cue type and their interaction on approach proportion, including model diagnostics (^R, bulk ESS and tail ESS). Parameters marked with an asterisk (*) indicate statistically credible effects where the 95% CI completely excludes zero.

parameter	estimate	est. error	l-95% CI	u-95% CI	Rhat	Bulk_ESS	Tail_ESS
intercept	–0.28	0.16	–0.60	0.04	1.00	5761	8719
day 2	0.22	0.24	–0.26	0.68	1.00	8791	11 033
day 3	0.14	0.19	–0.23	0.49	1.00	7461	11 165
day 4	0.20	0.21	–0.21	0.60	1.00	7926	10 424
day 5	0.23	0.22	–0.20	0.65	1.00	8273	11 462
day 6A *	**0.48**	**0.22**	**0.05**	**0.90**	**1.00**	**8640**	**11 174**
day 6B	0.34	0.21	–0.06	0.74	1.00	7612	10 297
cue threateningday1	0.40	0.21	0.00	0.80	1.00	5788	8684
day 2:cue threatening	–0.50	0.31	–1.09	0.11	1.00	9353	10 864
day 3:cue threatening	–0.50	0.28	–1.06	0.06	1.00	8918	11 285
day 4:cue threatening	–0.40	0.26	–0.92	0.11	1.00	8021	11 035
day 5:cue threatening *	–**0.64**	**0.28**	–**1.18**	–**0.09**	**1.00**	**8327**	**10 548**
day 6A:cue threatening *	–**0.67**	**0.30**	–**1.24**	–**0.08**	**1.00**	**9353**	**11 268**
day 6B:cue threatening *	–**0.88**	**0.28**	–**1.43**	–**0.31**	**1.00**	**8630**	**9816**

To interpret this interaction, we examined estimated marginal means, which represent model-predicted effects averaged over other predictors.

#### 3.1.1. What was the difference in the approach proportion within a day, between the positive and threatening cues?

On day 1, before any cue was given, approach tended to be lower in groups assigned to the positive cue than in those assigned to the threatening cue, although the 95% HPD interval just included zero (median estimate = −0.10; 95% HPD: −0.20, 0.0003; figure 2a). There were no credible differences on any other day.

**Figure 2.**
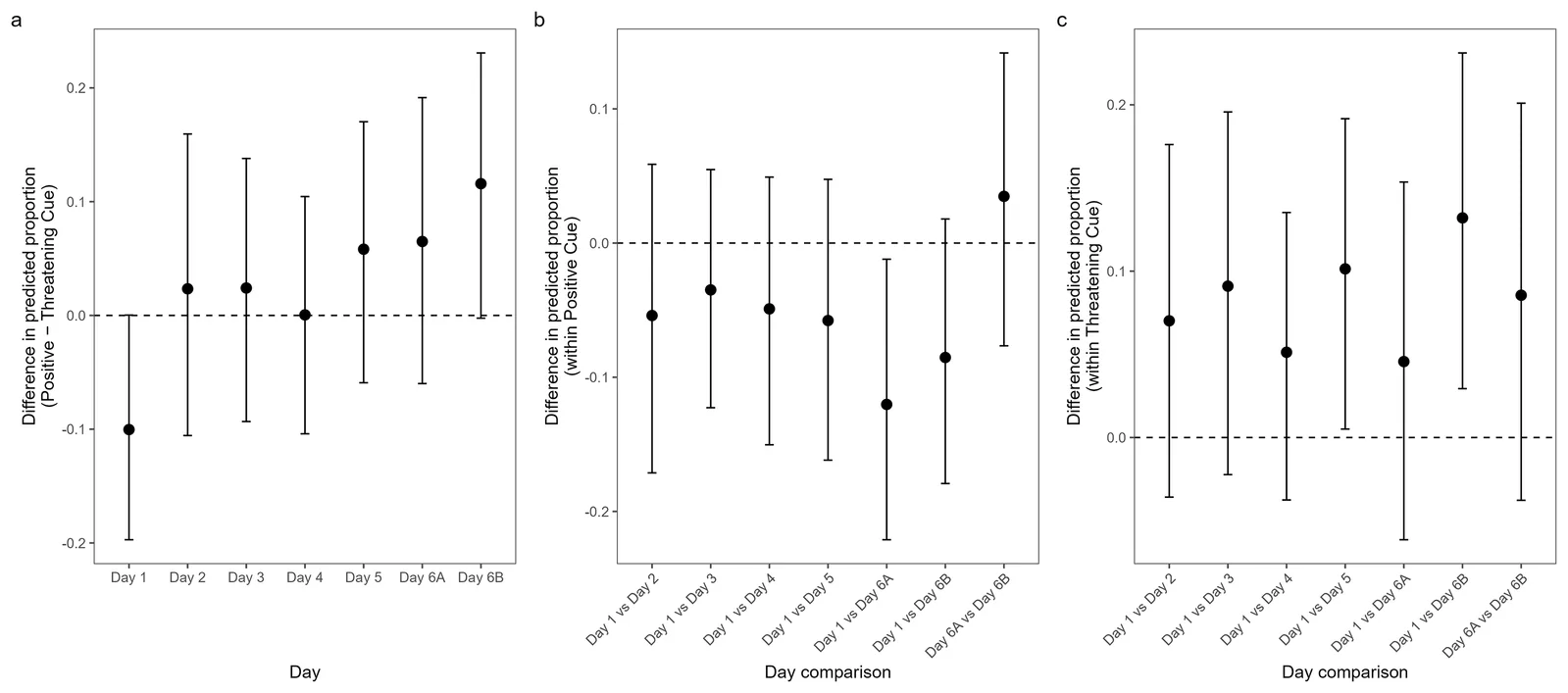
Difference in predicted proportion for (a) between-cue contrasts (positive—threatening cue) across days, (b) within-positive cue contrasts between selected day comparisons (relative to day 1 and between days 6A and 6B) and (c) within-threatening cue contrasts showing analogous day-to-day changes under the threatening cue condition. Points represent posterior medians and error bars indicate 95% HPD. The dashed horizontal line at zero indicates no difference.

#### 3.1.2. What was the difference in approach proportion across days within the positive cue?

Within the positive cue groups, approach proportion was higher on day 6A than on day 1 (day 1–6A: median estimate = −0.12; 95% HPD: −0.22, −0.01; figure 2b); no other day differed from day 1.

#### 3.1.3. What was the difference in approach proportion across days within the threatening cue?

Approach proportion on day 1 was higher than on day 5 (median estimate = 0.10; 95% HPD: 0.01, 0.19) and day 6B (median estimate = 0.13; 95% HPD: 0.03, 0.23; figure 2c). All the pairwise comparisons are presented in table S1 in the supplementary material.

### 3.2. What factors affected the approach latency during the approachability test?

The lognormal mixed-effects survival model revealed clear effects of test day, cue type, their interaction and sex on approach latency (table 2).

**Table 2.** Posterior parameter estimates (posterior mean (estimate), posterior standard deviation (est.Error) and 95% credible intervals) from the Bayesian regression model assessing the effects of day, cue type, sex and their interaction on the response variable, including model diagnostics (R̂, bulk ESS and tail ESS). Parameters marked with an asterisk (*) indicate statistically credible effects where the 95% CI completely excludes zero.

parameter	estimate	est.Error	l-95% CI	u-95% CI	Rhat	Bulk_ESS	Tail_ESS
intercept *	**4.51**	**0.27**	**3.98**	**5.04**	**1.00**	**6619**	**9416**
DAYDay2	–0.09	0.26	–0.61	0.42	1.00	7235	10 909
DAYDay3	–0.26	0.26	–0.76	0.25	1.00	7408	10 638
DAYDay4 *	–**0.80**	**0.25**	–**1.28**	–**0.32**	**1.00**	**7131**	**10 045**
DAYDay5 *	–**0.69**	**0.25**	–**1.18**	–**0.20**	**1.00**	**7120**	**10 254**
DAYDay6A	–0.28	0.26	–0.80	0.23	1.00	7520	9834
DAYDay6B	–0.50	0.26	–1.00	0.01	1.00	6676	10 080
cuethreateningDay1 *	–**0.76**	**0.33**	–**1.41**	–**0.10**	**1.00**	**6332**	**9097**
SEXM *	–**0.51**	**0.19**	–**0.88**	–**0.14**	**1.00**	**8847**	**11 972**
DAYDay2:cuethreatening *	**1.07**	**0.36**	**0.36**	**1.77**	**1.00**	**8975**	**11 894**
DAYDay3:cuethreatening *	**1.45**	**0.36**	**0.75**	**2.15**	**1.00**	**9249**	**11 659**
DAYDay4:cuethreatening *	**1.57**	**0.35**	**0.89**	**2.25**	**1.00**	**8898**	**10 976**
DAYDay5:cuethreatening *	**1.81**	**0.35**	**1.12**	**2.51**	**1.00**	**8990**	**11 490**
DAYDay6A:cuethreatening *	**0.89**	**0.35**	**0.20**	**1.58**	**1.00**	**9061**	**10 971**
DAYDay6B:cuethreatening *	**1.74**	**0.37**	**1.01**	**2.46**	**1.00**	**8565**	**11 138**

Estimated marginal means further clarified day- and cue-specific patterns.

#### 3.2.1. What was the difference in approach latency within day between the positive and threatening cues?

Day-specific contrasts between cue types showed that on day 1, approach latency was longer for the positive cue groups than the threatening cue ones (median estimate = 0.76; 95% HPD: 0.11, 1.41). From day 4 onward, this pattern reversed, with approach latency credibly longer under the threatening cue on day 4 (median estimate = −0.81; 95% HPD: −1.53, −0.06), day 5 (median estimate = −1.05; 95% HPD: −1.83, −0.28), and day 6B (median estimate = −0.97; 95% HPD: −1.74, −0.17; figure 3a).

**Figure 3.**
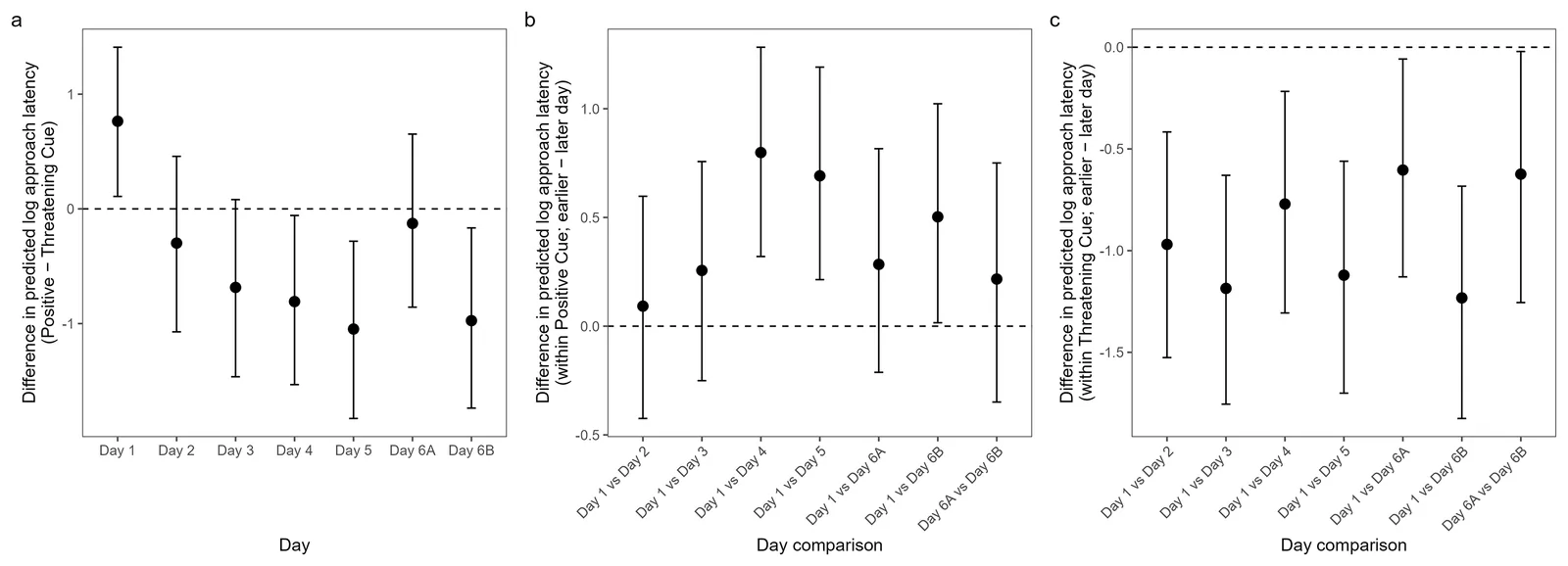
Differences in predicted log approach latency for (a) between-cue contrasts (positive—threatening cue) on each day, (b) within-positive cue contrasts relative to day 1 and between days 6A and 6B and (c) within-threatening cue contrasts relative to day 1 and between days 6A and 6B (earlier—later day). Points represent posterior medians and error bars indicate 95% HPD intervals. The dashed horizontal line at zero indicates no difference.

#### 3.2.2. What was the difference in approach latency across days within the positive cue?

Approach latencies on Day 1 were higher than those on Day 4 (median estimate = 0.80; 95% HPD: 0.32, 1.28), Day 5 (median estimate = 0.69; 95% HPD: 0.21, 1.19), and Day 6B (median estimate = 0.50; 95% HPD: 0.02, 1.02; figure 3b).

#### 3.2.3. What was the difference in approach latency across days within the threatening cue?

Approach latency was credibly lower on day 1 compared with subsequent days, including day 2 (median estimate = −0.97; 95% HPD: −1.53, −0.42), day 3 (median estimate = −1.19; 95% HPD: −1.75, −0.63), day 4 (median estimate = −0.77; 95% HPD: −1.31, −0.22), day 5 (median estimate = −1.12; 95% HPD: −1.70, −0.56), day 6A (median estimate = −0.60; 95% HPD: −1.13, −0.06) and day 6B (median estimate = −1.23; 95% HPD: −1.83, −0.68), indicating faster initial approach followed by longer latencies on later days. Latency was also shorter towards the unfamiliar experimenter on day 6A than towards the familiar experimenter on day 6B (day 6A–6B: median estimate = −0.62; 95% HPD: −1.26, −0.02; figure 3c). All pairwise comparisons are presented in table S2 in the supplementary material.

#### 3.2.4. What was the difference in approach latency between male and female dogs?

Female dogs showed higher approach latency compared with male dogs (median estimate = 0.51; 95% HPD: 0.15, 0.88).

### 3.3. What factors affected demeanour during the approachability test?

The model revealed credible effects of day and cue on dogs' demeanour during the approachability test (table 3).

**Table 3.** Posterior parameter estimates (posterior mean (estimate), posterior standard deviation (est.Error) and 95% credible intervals) from the Bayesian multinomial regression model assessing the effects of day, cue type, sex and their interaction on demeanour categories (aggressive, anxious and neutral and affiliative) during the approachability test, including model diagnostics (R̂, bulk ESS and tail ESS). ‘AG’, ‘AN’ and ‘N’ represent aggressive, anxious and neutral demeanours compared against the reference demeanour ‘AF’ or affiliative. Parameters marked with an asterisk (*) indicate statistically credible effects where the 95% CI completely excludes zero.

parameter	estimate	est.Error	l-95% CI	u-95% CI	Rhat	Bulk_ESS	Tail_ESS
muAG_Intercept *	**–2.27**	**0.48**	**–3.24**	**–1.37**	**1.00**	**11 499**	**12 059**
muAN_Intercept *	**–4.13**	**0.57**	**–5.30**	**–3.06**	**1.00**	**15 262**	**11 822**
muN_Intercept *	**0.99**	**0.30**	**0.40**	**1.59**	**1.00**	**10 304**	**11 655**
muAG_CUETHREATENINGDay1	0.50	0.51	**–**0.53	1.51	1.00	12 219	11 569
muAG_DAYDay2	**–**0.33	0.49	**–**1.32	0.60	1.00	17 229	12 364
muAG_DAYDay3	**–**0.85	0.52	**–**1.92	0.14	1.00	18 313	12 851
muAG_DAYDay4	**–**0.89	0.51	**–**1.94	0.08	1.00	19 386	12 023
muAG_DAYDay5 *	**–1.60**	**0.58**	**–2.76**	**–0.49**	**1.00**	**19 772**	**12 026**
muAG_DAYDay6A	**–**0.89	0.49	**–**1.87	0.04	1.00	19 355	12 326
muAG_DAYDay6B *	**–1.17**	**0.53**	**–2.25**	**–0.15**	**1.00**	**20 894**	**12 778**
muAG_SEXM	0.02	0.33	**–**0.64	0.68	1.00	18 103	11 829
muAG_CUETHREATENING:DAYDay2	0.33	0.59	**–**0.83	1.49	1.00	18 160	12 348
muAG_CUETHREATENING:DAYDay3	**–**0.07	0.64	**–**1.33	1.17	1.00	18 935	11 257
muAG_CUETHREATENING:DAYDay4	**–**0.36	0.64	**–**1.64	0.88	1.00	19 669	12 367
muAG_CUETHREATENING:DAYDay5	0.25	0.71	**–**1.15	1.62	1.00	20 202	12 812
muAG_CUETHREATENING:DAYDay6A	0.35	0.60	**–**0.81	1.52	1.00	20 186	12 260
muAG_CUETHREATENING:DAYDay6B	**–**0.19	0.71	**–**1.60	1.18	1.00	21 650	12 717
muAN_CUETHREATENINGDay1 *	**1.10**	**0.53**	**0.06**	**2.15**	**1.00**	**17 558**	**12 458**
muAN_DAYDay2	0.27	0.67	**–**1.07	1.56	1.00	18 657	12 630
muAN_DAYDay3	0.71	0.63	**–**0.54	1.93	1.00	18 675	11 013
muAN_DAYDay4	**–**0.04	0.68	**–**1.39	1.25	1.00	21 214	12 289
muAN_DAYDay5	**–**0.37	0.73	**–**1.85	1.00	1.00	22 704	12 082
muAN_DAYDay6A	**–**0.55	0.72	**–**1.98	0.81	1.00	21 799	12 624
muAN_DAYDay6B	0.43	0.64	**–**0.88	1.64	1.00	18 957	11 944
muAN_SEXM	**–**0.26	0.40	**–**1.04	0.51	1.00	23 954	12 504
muAN_CUETHREATENING:DAYDay2	**–**0.03	0.75	**–**1.51	1.41	1.00	17 738	11 678
muAN_CUETHREATENING:DAYDay3	0.90	0.67	**–**0.42	2.21	1.00	18 663	11 455
muAN_CUETHREATENING:DAYDay4	0.62	0.72	**–**0.78	2.06	1.00	21 357	12 239
muAN_CUETHREATENING:DAYDay5	0.04	0.80	**–**1.54	1.59	1.00	21 746	13 419
muAN_CUETHREATENING:DAYDay6A	**–**0.08	0.80	**–**1.67	1.46	1.00	21 838	12 465
muAN_CUETHREATENING:DAYDay6B	0.58	0.71	**–**0.81	1.98	1.00	20 198	11 944
muN_CUETHREATENINGDay1	**–**0.25	0.37	**–**0.98	0.48	1.00	10 357	10 419
muN_DAYDay2	0.25	0.31	**–**0.34	0.85	1.00	12 529	12 203
muN_DAYDay3	0.29	0.30	**–**0.29	0.87	1.00	12 151	12 716
muN_DAYDay4	**–**0.27	0.29	**–**0.83	0.29	1.00	12 820	13 016
muN_DAYDay5	**–**0.14	0.29	**–**0.71	0.43	1.00	12 946	12 514
muN_DAYDay6A *	**–0.89**	**0.30**	**–1.47**	**–0.31**	**1.00**	**13 317**	**11 961**
muN_DAYDay6B	**–**0.37	0.29	**–**0.95	0.21	1.00	13 696	12 857
muN_SEXM *	**–0.58**	**0.23**	**–1.04**	**–0.14**	**1.00**	**11 496**	**11 911**
muN_CUETHREATENING:DAYDay2	0.26	0.42	**–**0.56	1.07	1.00	14 442	12 297
muN_CUETHREATENING:DAYDay3	**–**0.07	0.41	**–**0.87	0.72	1.00	13 529	11 659
muN_CUETHREATENING:DAYDay4	0.45	0.40	**–**0.34	1.23	1.00	13 192	12 566
muN_CUETHREATENING:DAYDay5 *	**1.10**	**0.41**	**0.29**	**1.92**	**1.00**	**14 410**	**12 335**
muN_CUETHREATENING:DAYDay6A *	**1.15**	**0.40**	**0.36**	**1.95**	**1.00**	**13 231**	**11 999**
muN_CUETHREATENING:DAYDay6B *	**1.42**	**0.41**	**0.60**	**2.22**	**1.00**	**14 017**	**12 115**

The results of pairwise contrasts are described below:

#### 3.3.1. What was the difference in the approachability test demeanour within a day, between the positive and threatening cues?

Estimated marginal contrasts of predicted probabilities indicated differences between cue types for some demeanour categories on specific days. Anxious demeanour was higher under the threatening cue than under the positive cue on day 3 (median estimate = 0.06, 95% HPD: 0.01, 0.12). Affiliative demeanour was less likely under the threatening cue on day 5 (median estimate = −0.16, 95% HPD: −0.34, −0.004), day 6A (median estimate = −0.21, 95% HPD: −0.39, −0.03) and day 6B (median estimate = −0.22, 95% HPD: −0.38, −0.06). By contrast, neutral demeanour was more likely under the threatening cue on day 6A (median estimate = 0.19, 95% HPD: 0.001, 0.39) and day 6B (median estimate = 0.21, 95% HPD: 0.04, 0.39; figure 4a). Overall, these results suggest that while early responses to cue type were largely similar, dogs increasingly showed reduced affiliative demeanour and increased neutral demeanour under the threatening cue with repeated exposure. The results of all pairwise contrasts are presented in table S3 in the supplementary material.

**Figure 4.**
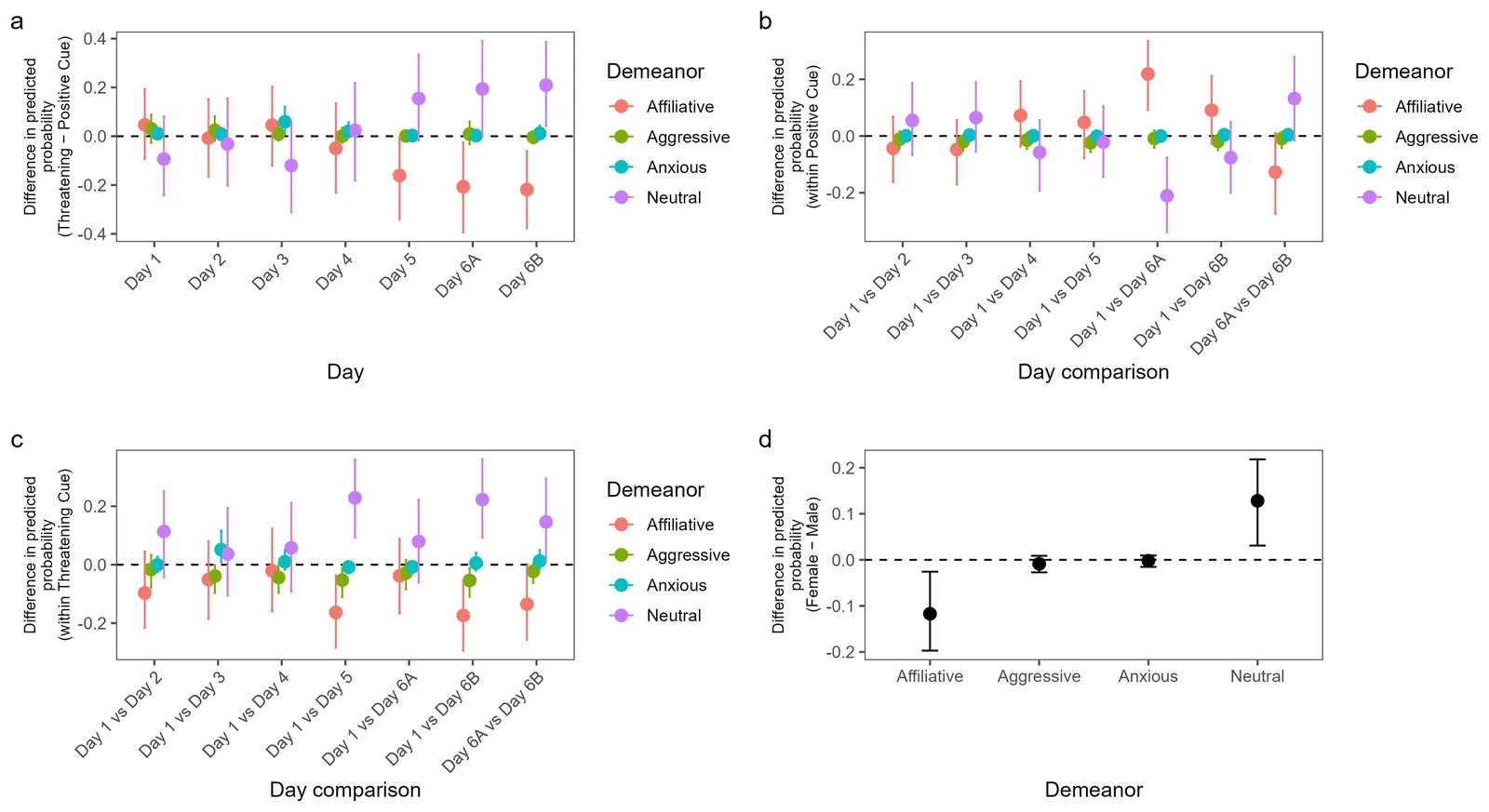
Difference in predicted probability for the different demeanours during the approachability test: (a) between-cue contrasts (threatening—positive cue) across days, (b) within-positive cue contrasts between selected day comparisons (relative to day 1 and between days 6A and 6B), (c) within-threatening cue contrasts showing analogous day-to-day changes, and (d) between-sex contrasts (female–male). Points represent posterior medians, and error bars indicate 95% HPD. The dashed horizontal line at zero indicates no difference.

#### 3.3.2. What was the difference in approachability test demeanour across days within the positive cue?

Affiliative demeanour was higher on day 6A than on day 1 (median estimate = 0.22, 95% HPD: 0.09, 0.33). Aggressive demeanour decreased from day 1 to day 3 (median estimate = −0.02, 95% HPD: −0.05, −0.0001) and day 5 (median estimate = −0.02, 95% HPD: −0.06, −0.002), with no other credible differences across days. Anxious demeanour showed no credible changes across test days. Neutral demeanour was lower on day 6A than on day 1 (median estimate = −0.21, 95% HPD: −0.34, −0.08; figure 4b). Overall, under the positive cue, affiliative demeanour increased and neutral demeanour decreased towards the unfamiliar experimenter on day 6A, while aggressive demeanour declined slightly and anxious demeanour remained stable. The results of all pairwise contrasts are presented in table S4 in the supplementary material.

#### 3.3.3. What was the difference in approachability test demeanour across days within the threatening cue?

Affiliative demeanour declined, with lower probabilities on day 5 (median estimate = −0.16, 95% HPD: −0.28, −0.04) and day 6B (median estimate = −0.17, 95% HPD: −0.29, −0.06) relative to day 1, and was also lower on day 6B than on day 6A (median estimate = −0.13, 95% HPD: −0.26, −0.003). Aggressive demeanour was lower than on day 1 on day 3 (median estimate = −0.04, 95% HPD: −0.10, −0.001), day 5 (median estimate = −0.05, 95% HPD: −0.11, −0.01) and day 6B (median estimate = −0.05, 95% HPD: −0.11, −0.01). Anxious demeanour increased from day 1 to day 3 (median estimate = 0.05, 95% HPD: 0.01, 0.12). Neutral demeanour increased, with higher probabilities on day 5 (median estimate = 0.23, 95% HPD: 0.09, 0.36) and day 6B (median estimate = 0.22, 95% HPD: 0.09, 0.36) relative to day 1, and from day 6A to day 6B (median estimate = 0.15, 95% HPD: 0.01, 0.29; figure 4c). Overall, these results suggest that under the threatening cue, dogs showed a shift from affiliative and, to a lesser extent, aggressive behaviour towards neutral behaviour with repeated exposure, and that this shift was specific to the familiar experimenter. The results of all pairwise contrasts are presented in table S5 in the supplementary material.

#### 3.3.4. What was the difference in approachability test demeanour between male and female dogs?

Females showed a lower predicted probability of displaying affiliative demeanour (median difference = −0.12; 95% HPD: −0.20, −0.03) and a higher predicted probability of displaying neutral demeanour (median difference = 0.13; 95% HPD: 0.03, 0.22) than males. Anxious and aggressive demeanours did not differ credibly between sexes (figure 4d). The contrasts for all demeanour categories are presented in table S6 in the supplementary material.

### 3.4. What factors affected demeanour during the test phase?

The model revealed credible effects of day and cue on dogs' demeanour during the test phase (table 4).

**Table 4.** Posterior parameter estimates (posterior mean (estimate), posterior standard deviation (est.Error) and 95% credible intervals) from the Bayesian multinomial regression model assessing the effects of day, cue type, sex and their interaction on demeanour categories (aggressive, anxious and neutral and affiliative) during the test phase, including model diagnostics (R̂, bulk ESS and tail ESS). ‘AG’, ‘AN’ and ‘N’ represent aggressive, anxious and neutral demeanours compared against the reference demeanour ‘AF’ or affiliative. Parameters marked with an asterisk (*) indicate statistically credible effects where the 95% CI completely excludes zero.

parameter	estimate	est.Error	l-95% CI	u-95% CI	Rhat	Bulk_ESS	Tail_ESS
muAG_Intercept *	**–2.81**	**0.55**	**–3.92**	**–1.79**	**1.00**	**10 933**	**12 388**
muAN_Intercept *	**–3.68**	**0.65**	**–5.02**	**–2.46**	**1.00**	**16 872**	**12 294**
muN_Intercept *	**1.13**	**0.27**	**0.61**	**1.67**	**1.00**	**12 982**	**11 377**
muAG_CUETHREATENINGDay1 *	**1.49**	**0.54**	**0.41**	**2.54**	**1.00**	**15 338**	**12 860**
muAG_DAYDay2	0.65	0.54	**–**0.41	1.70	1.00	20 147	12 873
muAG_DAYDay3	**–**0.86	0.61	**–**2.09	0.28	1.00	20 483	12 064
muAG_DAYDay4	**–**0.51	0.58	**–**1.69	0.61	1.00	19 465	13 072
muAG_DAYDay5	**–**0.80	0.62	**–**2.03	0.39	1.00	20 809	12 412
muAG_SEXM	**–**0.11	0.38	**–**0.87	0.64	1.00	22 820	13 065
muAG_CUETHREATENING:DAYDay2	0.37	0.61	**–**0.80	1.57	1.00	20 232	12 519
muAG_CUETHREATENING:DAYDay3	0.32	0.67	**–**0.98	1.63	1.00	20 671	12 459
muAG_CUETHREATENING:DAYDay4	**–**0.64	0.68	**–**2.00	0.66	1.00	21 483	13 140
muAG_CUETHREATENING:DAYDay5	0.33	0.68	**–**1.00	1.67	1.00	20 991	13 048
muAN_CUETHREATENINGDay1 *	**1.50**	**0.61**	**0.29**	**2.69**	**1.00**	**17 600**	**13 215**
muAN_DAYDay2	**–**0.04	0.66	**–**1.36	1.23	1.00	23 274	12 796
muAN_DAYDay3	0.10	0.63	**–**1.12	1.32	1.00	21 191	12 768
muAN_DAYDay4	**–**0.52	0.66	**–**1.83	0.76	1.00	22 651	13 030
muAN_DAYDay5	**–**0.43	0.67	**–**1.79	0.88	1.00	22 648	13 124
muAN_SEXM	**–**0.03	0.44	**–**0.88	0.85	1.00	23 711	11 771
muAN_CUETHREATENING:DAYDay2	0.72	0.70	**–**0.65	2.10	1.00	22 704	12 985
muAN_CUETHREATENING:DAYDay3	0.42	0.68	**–**0.92	1.75	1.00	22 720	12 898
muAN_CUETHREATENING:DAYDay4	0.18	0.72	**–**1.22	1.60	1.00	21 463	12 339
muAN_CUETHREATENING:DAYDay5	0.23	0.74	**–**1.24	1.68	1.00	23 679	12 690
muN_CUETHREATENINGDay1	0.03	0.33	**–**0.62	0.67	1.00	11 904	11 737
muN_DAYDay2	0.42	0.31	**–**0.19	1.03	1.00	16 597	13 194
muN_DAYDay3	0.10	0.29	**–**0.47	0.67	1.00	14 844	13 215
muN_DAYDay4	**–**0.04	0.29	**–**0.61	0.54	1.00	15 883	13 949
muN_DAYDay5	0.26	0.30	**–**0.33	0.85	1.00	15 740	12 722
muN_SEXM	**–**0.14	0.20	**–**0.53	0.25	1.00	17 286	13 595
muN_CUETHREATENING:DAYDay2	0.36	0.45	**–**0.51	1.23	1.00	16 988	12 730
muN_CUETHREATENING:DAYDay3	0.19	0.42	**–**0.62	1.00	1.00	14 854	13 182
muN_CUETHREATENING:DAYDay4	0.36	0.41	**–**0.44	1.15	1.00	15 884	13 401
muN_CUETHREATENING:DAYDay5	0.50	0.43	**–**0.34	1.35	1.00	16 748	12 714

The results of pairwise contrasts are described below:

#### 3.4.1. What was the difference in test phase demeanour within day between the positive and threatening cues?

Aggressive demeanour was higher under the threatening cue than under the positive cue on day 1 (median estimate = 0.04, 95% HPD: 0.01, 0.10) and day 2 (median estimate = 0.06, 95% HPD: 0.004, 0.15), but did not differ between cue conditions on later days. No other demeanour category differed credibly between cue conditions on any day (figure 5a). The results of all contrasts are presented in table S7 in the supplementary material.

**Figure 5.**
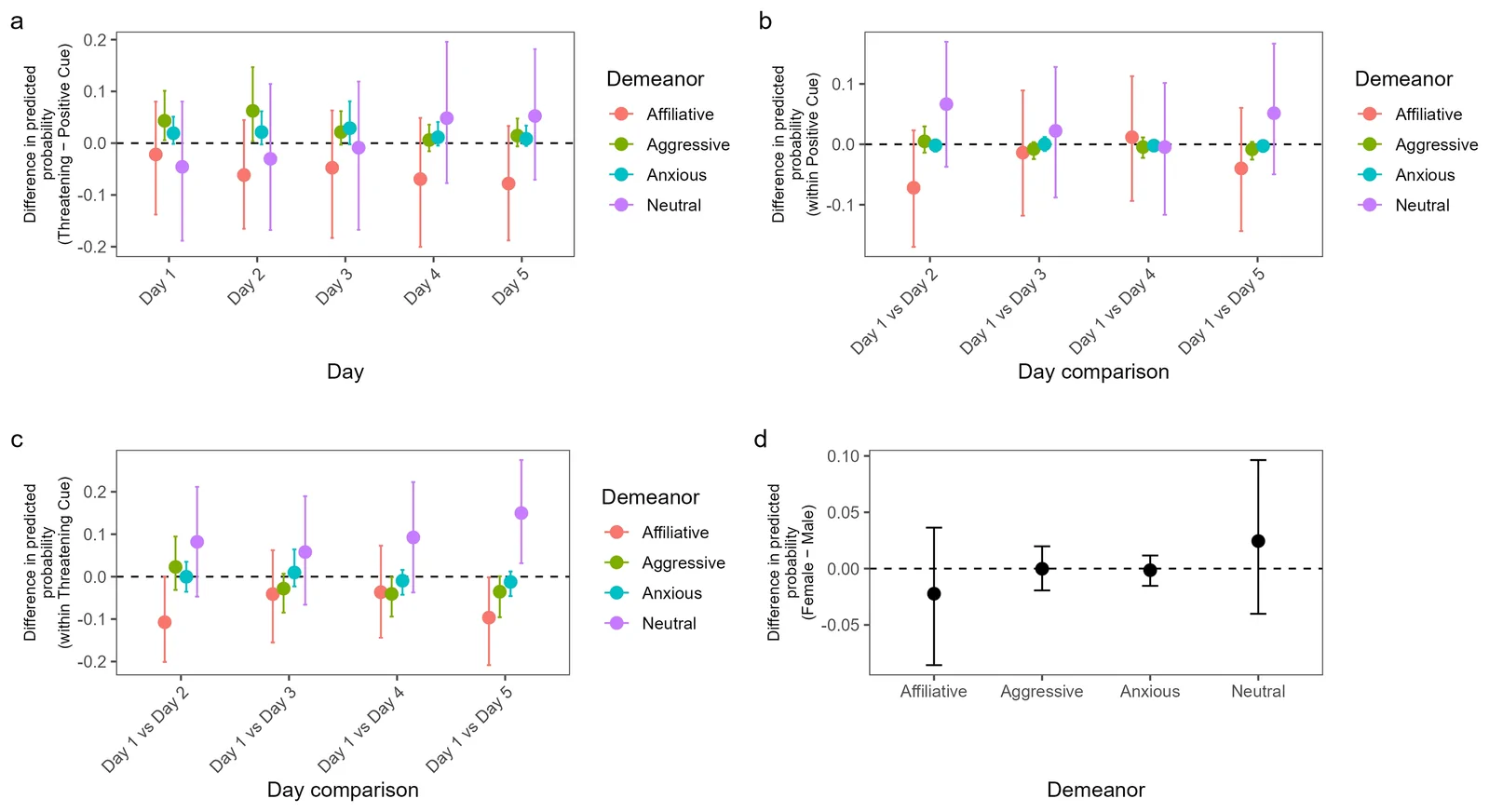
Difference in predicted probability for the different demeanours during the test phase: (a) between-cue contrasts (threatening—positive cue) across days, (b) within-positive cue contrasts between selected day comparisons (relative to day 1), (c) within-threatening cue contrasts showing analogous day-to-day changes, and (d) between-sex contrasts (female–male). Points represent posterior medians and error bars indicate 95% HPD. The dashed horizontal line at zero indicates no difference.

#### 3.4.2. What was the difference in test phase demeanour across days within the positive cue?

Within the positive cue condition, none of the demeanour categories showed credible changes across test days (figure 5b). The results of all pairwise contrasts are presented in table S8 in the supplementary material.

#### 3.4.3. What was the difference in test phase demeanour across days within the threatening cue?

Affiliative demeanour was lower on day 2 (median estimate = −0.11, 95% HPD: −0.20, −0.00015) and day 5 (median estimate = −0.10, 95% HPD: −0.21, −0.002) than on day 1. Aggressive demeanour was lower on day 4 than on day 1 (median estimate = −0.04, 95% HPD: −0.09, −0.001). Anxious demeanour showed no credible changes across test days. Neutral demeanour was higher on day 5 than on day 1 (median estimate = 0.15, 95% HPD: 0.03, 0.27; figure 5c). Overall, these results suggest that under the threatening cue, dogs' responses to the stick shifted from affiliative and aggressive towards neutral demeanour with repeated exposure, while anxious demeanour remained stable. The results of all pairwise contrasts are presented in table S9 in the supplementary material.

### 3.5. What was the difference in test phase demeanour between male and female dogs?

No demeanour category differed credibly between female and male dogs during the test phase (figure 5d). The contrasts for all demeanour categories are presented in table S10 in the supplementary material.

## 4. Discussion

The present study investigated how positive and threatening human cues influence sociability in Indian free-ranging dogs over repeated exposures, and whether these effects generalize to an unfamiliar experimenter. Free-ranging dogs in India live in close association with humans [29] and are therefore frequently exposed to both affiliative and aversive interactions, some of which can even be lethal [30,31]. Such experiences are likely to play a crucial role in shaping their behaviour towards humans [19]. The ability to evaluate and learn from human interactions is therefore highly adaptive, enabling these animals to maximize benefits from positive interactions while minimizing risks associated with potential threats [32]. One-time tests have previously examined the responses of FRDs to positive and threatening cues from unfamiliar humans [33,34]. The present study shows how dogs adjust their responses over repeated exposure to a cue from the same person (the familiar experimenter), and how this might affect their interactions with an unfamiliar person.

Our results provide mixed support for our four hypotheses. (a) The threatening cue reduced approachability towards the familiar experimenter: approach proportion decreased, and approach latency increased, over repeated interactions. (b) The positive cue reduced approach latency towards the familiar experimenter but did not increase approach proportion. (c) Behavioural changes emerged earlier under the threatening cue: approach latency differed from day 1, on day 2 and beyond under the threatening cue, but only from day 4 under the positive cue, though demeanour changes occurred from day three towards both cues. (d) Contrary to our prediction, both cues partly carried over to the unfamiliar experimenter, although on different measures, while several demeanour changes remained specific to the familiar experimenter.

On day 1, before any cue was administered, groups assigned to the threatening cue approached faster and tended to approach more often than groups assigned to the positive cue, although the difference in approach proportion was marginal. This difference appears to be driven by the distribution of extreme responses, with some threatening-cue groups showing complete approach and some positive-cue groups showing complete non-approach. Because the cue was delivered only after the approachability test and cue assignment was randomized, this baseline difference was not driven by the cue itself. However, it is important to note that the two groups, one assigned to the positive cue and the other to the threatening cue, started from different levels, which must be kept in mind when interpreting later within-cue changes.

Unexpectedly, groups exposed to the positive cue approached more on day 6A, when tested by the unfamiliar experimenter, than on day 1, but did not differ from day 1 on any other day with the familiar experimenter. Multiple explanations are worth considering. First, dogs vary in their approachability towards different experimenters [35], and the unfamiliar experimenter might simply have been more approachable. Second, because the positive-cue groups tended to start lower, part of this increase may reflect regression to the mean: on day 6A, their approach proportion rose to a level similar to that shown by the threatening-cue groups on day 1. Third, repeated exposure to food may promote a more general increase in willingness to approach humans [32]. Notably, approach proportion towards the unfamiliar experimenter did not differ between positive- and threatening-cue groups on day 6A, so the evidence that prior cue exposure shaped responses to the unfamiliar person should be treated with caution.

By contrast, the threatening-cue groups approached the familiar experimenter less often on later days (day 5 and day 6B) than on day 1. This indicates that repeated threatening interactions from the same person reduced approachability over time. Approach proportion towards the unfamiliar experimenter on day 6A, however, did not differ from day 1. This suggests that dogs maintained a baseline willingness to approach unfamiliar humans and did not extend reduced approach to other people, although, as mentioned below, they did approach the unfamiliar experimenter more slowly.

Approach latency refined these findings. On day 1, before any cue was administered, positive-cue groups showed longer approach latency than threatening-cue groups, again pointing to the baseline difference in approachability. From day 4 onward (days 4, 5 and 6B), this pattern reversed, with threatening-cue groups taking longer to approach the familiar experimenter than positive-cue groups. This provides the clearest evidence that cue valence shaped behaviour towards the familiar person. Within the positive-cue groups, approach latency decreased relative to day 1 on days 4, 5 and 6B. Latency towards the unfamiliar experimenter on day 6A, however, did not differ from day 1. The increased approach towards the unfamiliar experimenter was therefore not accompanied by a reduction in approach latency. Within the threatening-cue groups, approach latency increased relative to Day 1 on every subsequent day, including day 6A with the unfamiliar experimenter. On day 6, however, these dogs approached the unfamiliar experimenter faster than the familiar experimenter, indicating that caution was greatest towards the person who had delivered the threatening cue. The increase in latency occurred even on days when approach proportion did not change (days 2–4 and 6A), indicating that threatening interactions increased caution in approaching both the familiar and the unfamiliar experimenter. On day 6A, latency did not differ between the two cue groups, but because the groups started from different baselines, similar absolute levels can still reflect opposite changes within each group. Because dogs that did not approach were assigned the maximum latency, latency partly reflects whether dogs approached at all, not only how quickly approaching dogs did so. The growing number of non-approaching dogs on later days under the threatening cue therefore contributes to the longer latencies observed.

The timing in which these changes occurred is consistent with a negativity bias [22]. Latency increased from day 2 onward under the threatening cue but decreased only from day 4 onward under the positive cue. Similarly, approach proportion towards the familiar experimenter declined by day 5 under the threatening cue but never increased under the positive cue. However, the threatening-cue groups showed shorter latencies and tended to show higher approach on day 1, before any cue was given, and therefore had more room to decline.

Demeanour during the approachability test showed similar trends. Anxious demeanour was higher in the threatening-cue groups than in the positive-cue groups on day 3. Within the threatening-cue groups, anxious demeanour also increased from day 1 to day 3. Together, these results suggest that threatening interactions increased anxiety on the initial days of the experiment but that this effect declined later on. Affiliative demeanour was higher in the positive-cue groups than in the threatening-cue groups on days 5, 6A and 6B, but the source of this difference varied. On days 5 and 6B, it reflected a decline in affiliative demeanour under the threatening cue. On day 6A, it reflected an increase in affiliative demeanour under the positive cue, together with a decrease in neutral demeanour (so that neutral demeanour was also higher in the threatening-cue groups on day 6A), paralleling the increase in approach proportion towards the unfamiliar experimenter. Aggressive demeanour decreased in both cue conditions, from day 1 to days 3 and 5 under the positive cue and on days 3, 5 and 6B under the threatening cue, although these effects were small.

Importantly, within the threatening-cue groups, the decline in affiliative and aggressive demeanours, and the increase in neutral demeanour were evident towards the familiar experimenter (days 5 and 6B) but not towards the unfamiliar experimenter on day 6A. Moreover, on the same day, dogs showed higher neutral and lower affiliative demeanour, and took longer to approach, towards the familiar experimenter than towards the unfamiliar experimenter (day 6B versus day 6A). Because these two tests were only 5 minutes apart and followed the same procedure, this within-day difference is unlikely to reflect changes in time or context. Together, these results suggest that dogs discriminated the person associated with the threatening cue, in line with evidence that free-ranging dogs learn to recognize a rewarding person after only a few interactions [20]. Individual recognition of people has earlier been reported in pet dogs [36] and crows have been shown to remember threatening people [37].

During the test phase, aggressive demeanour was higher in the threatening-cue groups than in the positive-cue groups on days 1 and 2, but this difference disappeared thereafter. Other demeanours did not differ between cue conditions, and no changes across days were observed within the positive-cue groups. Within the threatening-cue groups, affiliative demeanour decreased on days 2 and 5 relative to day 1, aggressive demeanour decreased on day 4, and neutral demeanour increased on day 5. These results indicate a shift from affiliative and aggressive towards neutral responses with repeated exposure. This shift may reflect habituation: once dogs learned that the stick caused no actual harm, they responded to it more neutrally.

Sex differences were also evident. Averaged across days, female dogs showed longer approach latency, a lower probability of affiliative demeanour and a higher probability of neutral demeanour during the approachability test than males, suggesting greater caution. Anxious and aggressive demeanours did not differ between sexes, and no sex differences were observed during the test phase. These results contrast with those of Nandi *et al.* [35], who reported lower socialization index values for male dogs than for females. This discrepancy may reflect methodological differences. Nandi *et al.* [35] included only dogs that passed the approachability test on the first day and measured the intensity of affiliative behaviours, whereas the present study included all dogs regardless of whether they approached and recorded only their initial demeanour.

Some limitations of this study should be taken into account. First, E3 was present during all sessions, and E2 used the same vocalization and procedure as E1. Dogs may therefore have responded to the familiar situation rather than to the person, which would inflate apparent generalization on day 6A. Second, the experimenters were not counterbalanced, and day 6A always preceded day 6B, as we wanted to check the response to the unfamiliar experimenter. Finally, the distance at which the dogs were present away from the experimenter on the different days varied. Since the dogs were off leash, it was not possible to keep that constant. The distance was not taken into consideration while calculating the approach latency since it was difficult to estimate the distance from the videos.

Overall, both cues partly carried over to the unfamiliar experimenter, but on different measures. Dogs exposed to the positive cue approached the unfamiliar experimenter more often, without approaching faster. Dogs exposed to the threatening cue approached the unfamiliar experimenter as often as on day 1 but more slowly. However, direct comparisons between cue groups on day 6A showed no difference, and several demeanour changes under the threatening cue were specific to the familiar experimenter. Free-ranging dogs thus appear to update their behaviour primarily towards the individual associated with an outcome, while carrying over some general caution or willingness to approach. Maintaining a baseline willingness to approach unfamiliar humans while adjusting caution in response to threats may allow free-ranging dogs to exploit resource opportunities while limiting risk in environments where human behaviour is highly variable and unpredictable.

Our results have several implications for human-dog coexistence in urban environments. Since exposing dogs to threatening cues did not reduce their approach towards the unfamiliar person, stick-based intimidation is likely to protect only the person using it rather than making dogs avoid humans in general. Conversely, food provisioning by some people may increase approach towards others, which is important for public awareness about feeding practices and bite risk. By demonstrating how free-ranging dogs learn from different types of human interaction, this study can inform public awareness initiatives and the design of humane management programmes, and help reduce human-dog conflict.

## Supplementary Material



## Data Availability

Data supporting the results are provided in the supplementary material [38].
