# Supplementary material for: Operant conditioning in Indian free-ranging dogs: effects of positive and threatening cues on sociability
Source: R Soc Open Sci. 2026 Nov 11;13(11):261465. doi: 10.1098/rsos.261465 (PMC13752531; doi:10.1098/rsos.261465)
Supplement: Supplementary file 1 [file rsos.261465.zip › rsos.261465/rsos261465_si_004.docx]

**Operant Conditioning in Indian Free-Ranging Dogs: Effects of Positive and Threatening Cues on Sociability**

**Supplementary information**

**Figure S1.** Study sites

**Supplementary tables**

**Table S1.** Posterior summary of pairwise contrasts for approach proportion during the approachability test across days and cue conditions. Estimates are posterior medians of the difference in predicted approach proportion (first condition − second condition). Lower and upper HPD give the bounds of the 95% highest posterior density interval. Rows marked with an asterisk (*) and shown in bold indicate credible differences, where the 95% HPD interval excludes zero.

| **Contrast Comparison** | **Estimate** | **Lower HPD (95%)** | **Upper HPD (95%)** |
| --- | --- | --- | --- |
| Day 1 POSITIVE - Day 2 POSITIVE | -0.053987 | -0.17125 | 0.05871 |
| Day 1 POSITIVE - Day 3 POSITIVE | -0.034899 | -0.12270 | 0.05479 |
| Day 1 POSITIVE - Day 4 POSITIVE | -0.049030 | -0.15030 | 0.04916 |
| Day 1 POSITIVE - Day 5 POSITIVE | -0.057727 | -0.16177 | 0.04750 |
| **Day 1 POSITIVE - Day 6A POSITIVE *** | **-0.120239** | **-0.22107** | **-0.01203** |
| Day 1 POSITIVE - Day 6B POSITIVE | -0.085271 | -0.17918 | 0.01797 |
| Day 1 POSITIVE - Day 1 THREATENING | -0.100336 | -0.19715 | 0.00029 |
| Day 1 POSITIVE - Day 2 THREATENING | -0.030477 | -0.15302 | 0.08926 |
| Day 1 POSITIVE - Day 3 THREATENING | -0.010177 | -0.13229 | 0.11371 |
| Day 1 POSITIVE - Day 4 THREATENING | -0.049292 | -0.14766 | 0.05660 |
| Day 1 POSITIVE - Day 5 THREATENING | 0.000331 | -0.10572 | 0.11361 |
| Day 1 POSITIVE - Day 6A THREATENING | -0.054721 | -0.17834 | 0.06617 |
| Day 1 POSITIVE - Day 6B THREATENING | 0.031402 | -0.08330 | 0.14874 |
| Day 2 POSITIVE - Day 3 POSITIVE | 0.019480 | -0.09586 | 0.12817 |
| Day 2 POSITIVE - Day 4 POSITIVE | 0.005023 | -0.11689 | 0.12145 |
| Day 2 POSITIVE - Day 5 POSITIVE | -0.003521 | -0.12605 | 0.12010 |
| Day 2 POSITIVE - Day 6A POSITIVE | -0.066198 | -0.18678 | 0.06189 |
| Day 2 POSITIVE - Day 6B POSITIVE | -0.031225 | -0.14958 | 0.08822 |
| Day 2 POSITIVE - Day 1 THREATENING | -0.046426 | -0.17215 | 0.07438 |
| Day 2 POSITIVE - Day 2 THREATENING | 0.023415 | -0.10557 | 0.15949 |
| Day 2 POSITIVE - Day 3 THREATENING | 0.044363 | -0.09404 | 0.17868 |
| Day 2 POSITIVE - Day 4 THREATENING | 0.005039 | -0.11810 | 0.12280 |
| Day 2 POSITIVE - Day 5 THREATENING | 0.054352 | -0.07026 | 0.18048 |
| Day 2 POSITIVE - Day 6A THREATENING | -0.001100 | -0.13607 | 0.13727 |
| Day 2 POSITIVE - Day 6B THREATENING | 0.084971 | -0.04763 | 0.21487 |
| Day 3 POSITIVE - Day 4 POSITIVE | -0.014916 | -0.11427 | 0.07946 |
| Day 3 POSITIVE - Day 5 POSITIVE | -0.023306 | -0.12255 | 0.07753 |
| Day 3 POSITIVE - Day 6A POSITIVE | -0.085704 | -0.18670 | 0.01242 |
| Day 3 POSITIVE - Day 6B POSITIVE | -0.050521 | -0.14515 | 0.04255 |
| Day 3 POSITIVE - Day 1 THREATENING | -0.066384 | -0.16079 | 0.03438 |
| Day 3 POSITIVE - Day 2 THREATENING | 0.004013 | -0.10969 | 0.11837 |
| Day 3 POSITIVE - Day 3 THREATENING | 0.024115 | -0.09327 | 0.13797 |
| Day 3 POSITIVE - Day 4 THREATENING | -0.014186 | -0.11046 | 0.08175 |
| Day 3 POSITIVE - Day 5 THREATENING | 0.035090 | -0.06406 | 0.13935 |
| Day 3 POSITIVE - Day 6A THREATENING | -0.020682 | -0.13898 | 0.09231 |
| Day 3 POSITIVE - Day 6B THREATENING | 0.065759 | -0.04872 | 0.17117 |
| Day 4 POSITIVE - Day 5 POSITIVE | -0.007929 | -0.11463 | 0.10127 |
| Day 4 POSITIVE - Day 6A POSITIVE | -0.070496 | -0.17630 | 0.04134 |
| Day 4 POSITIVE - Day 6B POSITIVE | -0.036424 | -0.14174 | 0.06751 |
| Day 4 POSITIVE - Day 1 THREATENING | -0.051235 | -0.16330 | 0.05363 |
| Day 4 POSITIVE - Day 2 THREATENING | 0.019404 | -0.10960 | 0.13612 |
| Day 4 POSITIVE - Day 3 THREATENING | 0.039328 | -0.08912 | 0.16096 |
| Day 4 POSITIVE - Day 4 THREATENING | 0.000485 | -0.10404 | 0.10450 |
| Day 4 POSITIVE - Day 5 THREATENING | 0.050365 | -0.05846 | 0.16417 |
| Day 4 POSITIVE - Day 6A THREATENING | -0.005622 | -0.12801 | 0.11901 |
| Day 4 POSITIVE - Day 6B THREATENING | 0.080558 | -0.03934 | 0.19729 |
| Day 5 POSITIVE - Day 6A POSITIVE | -0.062629 | -0.17249 | 0.05257 |
| Day 5 POSITIVE - Day 6B POSITIVE | -0.027563 | -0.13348 | 0.08376 |
| Day 5 POSITIVE - Day 1 THREATENING | -0.042490 | -0.15477 | 0.07215 |
| Day 5 POSITIVE - Day 2 THREATENING | 0.027150 | -0.09770 | 0.15585 |
| Day 5 POSITIVE - Day 3 THREATENING | 0.047412 | -0.08838 | 0.16854 |
| Day 5 POSITIVE - Day 4 THREATENING | 0.008663 | -0.10349 | 0.11890 |
| Day 5 POSITIVE - Day 5 THREATENING | 0.058207 | -0.05917 | 0.17024 |
| Day 5 POSITIVE - Day 6A THREATENING | 0.002935 | -0.12743 | 0.13036 |
| Day 5 POSITIVE - Day 6B THREATENING | 0.089040 | -0.03200 | 0.21310 |
| Day 6A POSITIVE - Day 6B POSITIVE | 0.034820 | -0.07655 | 0.14168 |
| Day 6A POSITIVE - Day 1 THREATENING | 0.020268 | -0.09255 | 0.13272 |
| Day 6A POSITIVE - Day 2 THREATENING | 0.088869 | -0.03363 | 0.21874 |
| Day 6A POSITIVE - Day 3 THREATENING | 0.110172 | -0.01933 | 0.23532 |
| Day 6A POSITIVE - Day 4 THREATENING | 0.071200 | -0.03846 | 0.18271 |
| **Day 6A POSITIVE - Day 5 THREATENING *** | **0.120974** | **0.00578** | **0.23409** |
| Day 6A POSITIVE - Day 6A THREATENING | 0.064986 | -0.05988 | 0.19147 |
| **Day 6A POSITIVE - Day 6B THREATENING *** | **0.150493** | **0.02734** | **0.27094** |
| Day 6B POSITIVE - Day 1 THREATENING | -0.015111 | -0.11878 | 0.09385 |
| Day 6B POSITIVE - Day 2 THREATENING | 0.055043 | -0.06767 | 0.17512 |
| Day 6B POSITIVE - Day 3 THREATENING | 0.075019 | -0.05037 | 0.19348 |
| Day 6B POSITIVE - Day 4 THREATENING | 0.036126 | -0.06388 | 0.14178 |
| Day 6B POSITIVE - Day 5 THREATENING | 0.085025 | -0.02482 | 0.19376 |
| Day 6B POSITIVE - Day 6A THREATENING | 0.030307 | -0.09271 | 0.15250 |
| Day 6B POSITIVE - Day 6B THREATENING | 0.115813 | -0.00241 | 0.23072 |
| Day 1 THREATENING - Day 2 THREATENING | 0.070154 | -0.03584 | 0.17610 |
| Day 1 THREATENING - Day 3 THREATENING | 0.090983 | -0.02230 | 0.19571 |
| Day 1 THREATENING - Day 4 THREATENING | 0.051239 | -0.03756 | 0.13525 |
| **Day 1 THREATENING - Day 5 THREATENING *** | **0.101376** | **0.00509** | **0.19165** |
| Day 1 THREATENING - Day 6A THREATENING | 0.045638 | -0.06145 | 0.15358 |
| **Day 1 THREATENING - Day 6B THREATENING *** | **0.132013** | **0.02936** | **0.23116** |
| Day 2 THREATENING - Day 3 THREATENING | 0.020170 | -0.10127 | 0.14558 |
| Day 2 THREATENING - Day 4 THREATENING | -0.018959 | -0.12391 | 0.08590 |
| Day 2 THREATENING - Day 5 THREATENING | 0.030918 | -0.07552 | 0.14721 |
| Day 2 THREATENING - Day 6A THREATENING | -0.024603 | -0.14448 | 0.09838 |
| Day 2 THREATENING - Day 6B THREATENING | 0.061483 | -0.05633 | 0.17865 |
| Day 3 THREATENING - Day 4 THREATENING | -0.038599 | -0.14758 | 0.06883 |
| Day 3 THREATENING - Day 5 THREATENING | 0.011300 | -0.10208 | 0.12433 |
| Day 3 THREATENING - Day 6A THREATENING | -0.044516 | -0.16849 | 0.07896 |
| Day 3 THREATENING - Day 6B THREATENING | 0.041174 | -0.07664 | 0.16013 |
| Day 4 THREATENING - Day 5 THREATENING | 0.049280 | -0.04000 | 0.14261 |
| Day 4 THREATENING - Day 6A THREATENING | -0.005635 | -0.11363 | 0.09925 |
| Day 4 THREATENING - Day 6B THREATENING | 0.080268 | -0.02434 | 0.17859 |
| Day 5 THREATENING - Day 6A THREATENING | -0.054949 | -0.16331 | 0.06005 |
| Day 5 THREATENING - Day 6B THREATENING | 0.031148 | -0.07553 | 0.13805 |
| Day 6A THREATENING - Day 6B THREATENING | 0.085514 | -0.03775 | 0.20097 |

**Table S2.** Posterior summary of pairwise contrasts for approach latency during the approachability test across days and cue conditions. Estimates are posterior medians of the difference in predicted log approach latency (first condition − second condition). Lower and upper HPD give the bounds of the 95% highest posterior density interval. Rows marked with an asterisk (*) and shown in bold indicate credible differences, where the 95% HPD interval excludes zero.

| **Contrast Comparison** | **Estimate** | **Lower HPD (95%)** | **Upper HPD (95%)** |
| --- | --- | --- | --- |
| Day 1 POSITIVE - Day 2 POSITIVE | 0.092178 | -0.42447 | 0.59752 |
| Day 1 POSITIVE - Day 3 POSITIVE | 0.256311 | -0.25050 | 0.75717 |
| **Day 1 POSITIVE - Day 4 POSITIVE *** | **0.798580** | **0.32042** | **1.28294** |
| **Day 1 POSITIVE - Day 5 POSITIVE *** | **0.691536** | **0.21417** | **1.19145** |
| Day 1 POSITIVE - Day 6A POSITIVE | 0.284335 | -0.21220 | 0.81625 |
| **Day 1 POSITIVE - Day 6B POSITIVE *** | **0.502937** | **0.01582** | **1.02302** |
| **Day 1 POSITIVE - Day 1 THREATENING *** | **0.763849** | **0.10849** | **1.40917** |
| Day 1 POSITIVE - Day 2 THREATENING | -0.204348 | -0.98564 | 0.53530 |
| Day 1 POSITIVE - Day 3 THREATENING | -0.423224 | -1.18667 | 0.35442 |
| Day 1 POSITIVE - Day 4 THREATENING | -0.009385 | -0.77492 | 0.70873 |
| Day 1 POSITIVE - Day 5 THREATENING | -0.354671 | -1.13694 | 0.39233 |
| Day 1 POSITIVE - Day 6A THREATENING | 0.157814 | -0.57205 | 0.90451 |
| Day 1 POSITIVE - Day 6B THREATENING | -0.470073 | -1.24063 | 0.30396 |
| Day 2 POSITIVE - Day 3 POSITIVE | 0.167501 | -0.38763 | 0.73833 |
| **Day 2 POSITIVE - Day 4 POSITIVE *** | **0.703619** | **0.19356** | **1.25936** |
| **Day 2 POSITIVE - Day 5 POSITIVE *** | **0.598607** | **0.05219** | **1.13926** |
| Day 2 POSITIVE - Day 6A POSITIVE | 0.191205 | -0.39090 | 0.74014 |
| Day 2 POSITIVE - Day 6B POSITIVE | 0.408500 | -0.16488 | 0.95830 |
| Day 2 POSITIVE - Day 1 THREATENING | 0.667426 | -0.07876 | 1.36980 |
| Day 2 POSITIVE - Day 2 THREATENING | -0.298376 | -1.07199 | 0.45790 |
| Day 2 POSITIVE - Day 3 THREATENING | -0.518736 | -1.32195 | 0.24649 |
| Day 2 POSITIVE - Day 4 THREATENING | -0.104218 | -0.87585 | 0.67031 |
| Day 2 POSITIVE - Day 5 THREATENING | -0.453217 | -1.25580 | 0.33446 |
| Day 2 POSITIVE - Day 6A THREATENING | 0.063775 | -0.67262 | 0.85834 |
| Day 2 POSITIVE - Day 6B THREATENING | -0.564278 | -1.36649 | 0.23593 |
| **Day 3 POSITIVE - Day 4 POSITIVE *** | **0.539705** | **0.00516** | **1.06663** |
| Day 3 POSITIVE - Day 5 POSITIVE | 0.433245 | -0.09555 | 0.94900 |
| Day 3 POSITIVE - Day 6A POSITIVE | 0.023761 | -0.52741 | 0.57261 |
| Day 3 POSITIVE - Day 6B POSITIVE | 0.244787 | -0.29092 | 0.78919 |
| Day 3 POSITIVE - Day 1 THREATENING | 0.507870 | -0.18858 | 1.24289 |
| Day 3 POSITIVE - Day 2 THREATENING | -0.463291 | -1.25781 | 0.28957 |
| Day 3 POSITIVE - Day 3 THREATENING | -0.684651 | -1.46331 | 0.08088 |
| Day 3 POSITIVE - Day 4 THREATENING | -0.268861 | -1.04280 | 0.47991 |
| Day 3 POSITIVE - Day 5 THREATENING | -0.615498 | -1.39095 | 0.18018 |
| Day 3 POSITIVE - Day 6A THREATENING | -0.098560 | -0.84507 | 0.65502 |
| Day 3 POSITIVE - Day 6B THREATENING | -0.726842 | -1.51701 | 0.05377 |
| Day 4 POSITIVE - Day 5 POSITIVE | -0.105874 | -0.60898 | 0.39549 |
| Day 4 POSITIVE - Day 6A POSITIVE | -0.513960 | -1.01909 | 0.02293 |
| Day 4 POSITIVE - Day 6B POSITIVE | -0.295491 | -0.84263 | 0.22859 |
| Day 4 POSITIVE - Day 1 THREATENING | -0.033902 | -0.73245 | 0.65873 |
| **Day 4 POSITIVE - Day 2 THREATENING *** | **-1.007831** | **-1.74402** | **-0.21868** |
| **Day 4 POSITIVE - Day 3 THREATENING *** | **-1.222946** | **-2.00172** | **-0.45567** |
| **Day 4 POSITIVE - Day 4 THREATENING *** | **-0.808102** | **-1.53205** | **-0.05752** |
| **Day 4 POSITIVE - Day 5 THREATENING *** | **-1.158649** | **-1.93953** | **-0.39076** |
| Day 4 POSITIVE - Day 6A THREATENING | -0.640181 | -1.37347 | 0.09586 |
| **Day 4 POSITIVE - Day 6B THREATENING *** | **-1.266553** | **-2.04729** | **-0.48319** |
| Day 5 POSITIVE - Day 6A POSITIVE | -0.405229 | -0.92577 | 0.12711 |
| Day 5 POSITIVE - Day 6B POSITIVE | -0.185782 | -0.71498 | 0.33583 |
| Day 5 POSITIVE - Day 1 THREATENING | 0.072817 | -0.62122 | 0.78761 |
| **Day 5 POSITIVE - Day 2 THREATENING *** | **-0.901510** | **-1.64262** | **-0.11602** |
| **Day 5 POSITIVE - Day 3 THREATENING *** | **-1.115927** | **-1.87354** | **-0.33249** |
| Day 5 POSITIVE - Day 4 THREATENING | -0.697864 | -1.45115 | 0.04212 |
| **Day 5 POSITIVE - Day 5 THREATENING *** | **-1.047527** | **-1.82716** | **-0.28209** |
| Day 5 POSITIVE - Day 6A THREATENING | -0.534146 | -1.27362 | 0.22366 |
| **Day 5 POSITIVE - Day 6B THREATENING *** | **-1.157367** | **-1.94417** | **-0.39570** |
| Day 6A POSITIVE - Day 6B POSITIVE | 0.216794 | -0.34889 | 0.75055 |
| Day 6A POSITIVE - Day 1 THREATENING | 0.477156 | -0.23672 | 1.18713 |
| Day 6A POSITIVE - Day 2 THREATENING | -0.494593 | -1.25201 | 0.29755 |
| Day 6A POSITIVE - Day 3 THREATENING | -0.711349 | -1.49106 | 0.08948 |
| Day 6A POSITIVE - Day 4 THREATENING | -0.297155 | -1.06604 | 0.46140 |
| Day 6A POSITIVE - Day 5 THREATENING | -0.643739 | -1.45041 | 0.13548 |
| Day 6A POSITIVE - Day 6A THREATENING | -0.126774 | -0.85682 | 0.65199 |
| Day 6A POSITIVE - Day 6B THREATENING | -0.754585 | -1.54563 | 0.03488 |
| Day 6B POSITIVE - Day 1 THREATENING | 0.261705 | -0.45948 | 0.95958 |
| Day 6B POSITIVE - Day 2 THREATENING | -0.712645 | -1.50011 | 0.04151 |
| **Day 6B POSITIVE - Day 3 THREATENING *** | **-0.926972** | **-1.73062** | **-0.16571** |
| Day 6B POSITIVE - Day 4 THREATENING | -0.513864 | -1.26850 | 0.25168 |
| **Day 6B POSITIVE - Day 5 THREATENING *** | **-0.860742** | **-1.62295** | **-0.05751** |
| Day 6B POSITIVE - Day 6A THREATENING | -0.350093 | -1.09805 | 0.41430 |
| **Day 6B POSITIVE - Day 6B THREATENING *** | **-0.974309** | **-1.73674** | **-0.16590** |
| **Day 1 THREATENING - Day 2 THREATENING *** | **-0.969274** | **-1.52577** | **-0.41650** |
| **Day 1 THREATENING - Day 3 THREATENING *** | **-1.185690** | **-1.75471** | **-0.62970** |
| **Day 1 THREATENING - Day 4 THREATENING *** | **-0.770689** | **-1.30649** | **-0.21715** |
| **Day 1 THREATENING - Day 5 THREATENING *** | **-1.120751** | **-1.70071** | **-0.56045** |
| **Day 1 THREATENING - Day 6A THREATENING *** | **-0.603785** | **-1.12895** | **-0.05801** |
| **Day 1 THREATENING - Day 6B THREATENING *** | **-1.232442** | **-1.82520** | **-0.68308** |
| Day 2 THREATENING - Day 3 THREATENING | -0.218903 | -0.86341 | 0.42383 |
| Day 2 THREATENING - Day 4 THREATENING | 0.197516 | -0.40416 | 0.82253 |
| Day 2 THREATENING - Day 5 THREATENING | -0.148715 | -0.80572 | 0.49840 |
| Day 2 THREATENING - Day 6A THREATENING | 0.368654 | -0.22355 | 0.99660 |
| Day 2 THREATENING - Day 6B THREATENING | -0.260274 | -0.92593 | 0.36861 |
| Day 3 THREATENING - Day 4 THREATENING | 0.417577 | -0.20174 | 1.05678 |
| Day 3 THREATENING - Day 5 THREATENING | 0.072602 | -0.57802 | 0.70868 |
| Day 3 THREATENING - Day 6A THREATENING | 0.581773 | -0.03511 | 1.20397 |
| Day 3 THREATENING - Day 6B THREATENING | -0.043698 | -0.70444 | 0.59251 |
| Day 4 THREATENING - Day 5 THREATENING | -0.349342 | -0.99302 | 0.25265 |
| Day 4 THREATENING - Day 6A THREATENING | 0.170191 | -0.42551 | 0.75957 |
| Day 4 THREATENING - Day 6B THREATENING | -0.458566 | -1.09768 | 0.17621 |
| Day 5 THREATENING - Day 6A THREATENING | 0.514582 | -0.10396 | 1.15337 |
| Day 5 THREATENING - Day 6B THREATENING | -0.110379 | -0.77278 | 0.55079 |
| **Day 6A THREATENING - Day 6B THREATENING *** | **-0.623926** | **-1.25529** | **-0.02100** |

**Table S3.** Posterior summary of contrasts in predicted demeanor probabilities between cue conditions (threatening − positive) on each day of the approachability test. Estimates are posterior medians. AF = affiliative, AG = aggressive, AN = anxious, N = neutral. Lower and upper HPD give the bounds of the 95% highest posterior density interval. Rows marked with an asterisk (*) and shown in bold indicate credible differences, where the 95% HPD interval excludes zero.

| **Day** | **Demeanor** | **Median** | **Lower HPD** | **Upper HPD** |
| --- | --- | --- | --- | --- |
| Day 1 | AF | 0.046797 | -0.092447 | 0.193999 |
| Day 1 | AG | 0.030405 | -0.027622 | 0.088293 |
| Day 1 | AN | 0.010679 | -0.000876 | 0.029351 |
| Day 1 | N | -0.093246 | -0.242716 | 0.081656 |
| Day 2 | AF | -0.007428 | -0.166769 | 0.152083 |
| Day 2 | AG | 0.024578 | -0.01887 | 0.082159 |
| Day 2 | AN | 0.008634 | -0.007964 | 0.034189 |
| Day 2 | N | -0.032185 | -0.202663 | 0.155494 |
| Day 3 | AF | 0.045591 | -0.121129 | 0.202914 |
| Day 3 | AG | 0.009487 | -0.018305 | 0.046638 |
| **Day 3** | **AN *** | **0.059092** | **0.010576** | **0.120887** |
| Day 3 | N | -0.119908 | -0.311573 | 0.061382 |
| Day 4 | AF | -0.049357 | -0.231702 | 0.135114 |
| Day 4 | AG | 0.00056 | -0.026921 | 0.035257 |
| Day 4 | AN | 0.020014 | -0.00183 | 0.057276 |
| Day 4 | N | 0.024426 | -0.181391 | 0.218324 |
| **Day 5** | **AF *** | **-0.16078** | **-0.340706** | **-0.003584** |
| Day 5 | AG | 0.001294 | -0.016615 | 0.022805 |
| Day 5 | AN | 0.00225 | -0.007951 | 0.02063 |
| Day 5 | N | 0.154765 | -0.017572 | 0.334578 |
| **Day 6A** | **AF *** | **-0.207125** | **-0.394025** | **-0.025215** |
| Day 6A | AG | 0.008914 | -0.033055 | 0.05958 |
| Day 6A | AN | 0.002836 | -0.009726 | 0.021981 |
| **Day 6A** | **N *** | **0.193833** | **0.000692** | **0.389885** |
| **Day 6B** | **AF *** | **-0.218787** | **-0.377154** | **-0.061058** |
| Day 6B | AG | -0.004068 | -0.029495 | 0.017072 |
| Day 6B | AN | 0.011646 | -0.013195 | 0.044216 |
| **Day 6B** | **N *** | **0.20932** | **0.041973** | **0.386393** |

**Table S4.** Posterior summary of pairwise contrasts in predicted demeanor probabilities across days of the approachability test within the positive cue condition (Day (B) − Day (A)). Estimates are posterior medians. AF = affiliative, AG = aggressive, AN = anxious, N = neutral. Lower and upper HPD give the bounds of the 95% highest posterior density interval. Rows marked with an asterisk (*) and shown in bold indicate credible differences, where the 95% HPD interval excludes zero.

| **Demeanor** | **Day (A)** | **Day (B)** | **Median** | **Lower HPD** | **Upper HPD** |
| --- | --- | --- | --- | --- | --- |
| AF | Day 1 | Day 2 | -0.04306 | -0.162548 | 0.068087 |
| AF | Day 1 | Day 3 | -0.047399 | -0.170942 | 0.056603 |
| AF | Day 1 | Day 4 | 0.07253 | -0.036992 | 0.19381 |
| AF | Day 1 | Day 5 | 0.048021 | -0.079232 | 0.15866 |
| **AF *** | **Day 1** | **Day 6A** | **0.218788** | **0.091868** | **0.33475** |
| AF | Day 1 | Day 6B | 0.091054 | -0.027265 | 0.212048 |
| AF | Day 2 | Day 3 | -0.002739 | -0.13044 | 0.110837 |
| AF | Day 2 | Day 4 | 0.115846 | -0.014832 | 0.24238 |
| AF | Day 2 | Day 5 | 0.092897 | -0.033842 | 0.229719 |
| **AF *** | **Day 2** | **Day 6A** | **0.262403** | **0.128025** | **0.394968** |
| AF | Day 2 | Day 6B | 0.135098 | -0.000427 | 0.273546 |
| AF | Day 3 | Day 4 | 0.121581 | -0.00543 | 0.252685 |
| AF | Day 3 | Day 5 | 0.097382 | -0.039489 | 0.216259 |
| **AF *** | **Day 3** | **Day 6A** | **0.264285** | **0.123377** | **0.394798** |
| **AF *** | **Day 3** | **Day 6B** | **0.139729** | **0.009452** | **0.273629** |
| AF | Day 4 | Day 5 | -0.025451 | -0.163282 | 0.112619 |
| AF | Day 4 | Day 6A | 0.14268 | -0.000982 | 0.287426 |
| AF | Day 4 | Day 6B | 0.017368 | -0.1201 | 0.159847 |
| **AF *** | **Day 5** | **Day 6A** | **0.169461** | **0.025365** | **0.305563** |
| AF | Day 5 | Day 6B | 0.043764 | -0.096267 | 0.192338 |
| AF | Day 6A | Day 6B | -0.127208 | -0.274367 | 0.010473 |
| AG | Day 1 | Day 2 | -0.011547 | -0.044223 | 0.016445 |
| **AG *** | **Day 1** | **Day 3** | **-0.0196** | **-0.052202** | **-0.000096** |
| AG | Day 1 | Day 4 | -0.014664 | -0.045556 | 0.011301 |
| **AG *** | **Day 1** | **Day 5** | **-0.023717** | **-0.056796** | **-0.002078** |
| AG | Day 1 | Day 6A | -0.008885 | -0.041896 | 0.019276 |
| AG | Day 1 | Day 6B | -0.018445 | -0.049735 | 0.005294 |
| AG | Day 2 | Day 3 | -0.007343 | -0.035265 | 0.015422 |
| AG | Day 2 | Day 4 | -0.003052 | -0.029737 | 0.025303 |
| AG | Day 2 | Day 5 | -0.011672 | -0.039705 | 0.00555 |
| AG | Day 2 | Day 6A | 0.002547 | -0.02567 | 0.034753 |
| AG | Day 2 | Day 6B | -0.006499 | -0.033828 | 0.021352 |
| AG | Day 3 | Day 4 | 0.004394 | -0.017235 | 0.030448 |
| AG | Day 3 | Day 5 | -0.003702 | -0.023263 | 0.013589 |
| AG | Day 3 | Day 6A | 0.010385 | -0.009654 | 0.044991 |
| AG | Day 3 | Day 6B | 0.000921 | -0.019398 | 0.025537 |
| AG | Day 4 | Day 5 | -0.008288 | -0.034824 | 0.00887 |
| AG | Day 4 | Day 6A | 0.005563 | -0.02123 | 0.040399 |
| AG | Day 4 | Day 6B | -0.003449 | -0.029417 | 0.020347 |
| AG | Day 5 | Day 6A | 0.014366 | -0.005059 | 0.047307 |
| AG | Day 5 | Day 6B | 0.004861 | -0.011684 | 0.028927 |
| AG | Day 6A | Day 6B | -0.009092 | -0.042539 | 0.015158 |
| AN | Day 1 | Day 2 | 0.00053 | -0.006733 | 0.010559 |
| AN | Day 1 | Day 3 | 0.003154 | -0.00474 | 0.018145 |
| AN | Day 1 | Day 4 | 0.00076 | -0.005792 | 0.012339 |
| AN | Day 1 | Day 5 | -0.000871 | -0.00911 | 0.007726 |
| AN | Day 1 | Day 6A | -0.000012 | -0.008325 | 0.009959 |
| AN | Day 1 | Day 6B | 0.004201 | -0.004311 | 0.021663 |
| AN | Day 2 | Day 3 | 0.002422 | -0.010063 | 0.018406 |
| AN | Day 2 | Day 4 | 0.000268 | -0.012652 | 0.013369 |
| AN | Day 2 | Day 5 | -0.001407 | -0.014523 | 0.009071 |
| AN | Day 2 | Day 6A | -0.000509 | -0.013551 | 0.011776 |
| AN | Day 2 | Day 6B | 0.003604 | -0.010318 | 0.022007 |
| AN | Day 3 | Day 4 | -0.002232 | -0.018725 | 0.01149 |
| AN | Day 3 | Day 5 | -0.003902 | -0.020296 | 0.008428 |
| AN | Day 3 | Day 6A | -0.003058 | -0.020518 | 0.009455 |
| AN | Day 3 | Day 6B | 0.001249 | -0.017538 | 0.018988 |
| AN | Day 4 | Day 5 | -0.001557 | -0.013254 | 0.009574 |
| AN | Day 4 | Day 6A | -0.000866 | -0.014122 | 0.011713 |
| AN | Day 4 | Day 6B | 0.003369 | -0.010929 | 0.021895 |
| AN | Day 5 | Day 6A | 0.000788 | -0.009758 | 0.013835 |
| AN | Day 5 | Day 6B | 0.005045 | -0.00613 | 0.026595 |
| AN | Day 6A | Day 6B | 0.004178 | -0.012566 | 0.020954 |
| N | Day 1 | Day 2 | 0.05472 | -0.067856 | 0.186176 |
| N | Day 1 | Day 3 | 0.065205 | -0.05589 | 0.190699 |
| N | Day 1 | Day 4 | -0.057743 | -0.192651 | 0.056765 |
| N | Day 1 | Day 5 | -0.021386 | -0.144852 | 0.104973 |
| **N *** | **Day 1** | **Day 6A** | **-0.210822** | **-0.339852** | **-0.0756** |
| N | Day 1 | Day 6B | -0.076261 | -0.200339 | 0.050643 |
| N | Day 2 | Day 3 | 0.0082 | -0.122302 | 0.135178 |
| N | Day 2 | Day 4 | -0.114034 | -0.246915 | 0.023816 |
| N | Day 2 | Day 5 | -0.076951 | -0.202609 | 0.075078 |
| **N *** | **Day 2** | **Day 6A** | **-0.264066** | **-0.391734** | **-0.109131** |
| N | Day 2 | Day 6B | -0.132201 | -0.277785 | 0.007722 |
| N | Day 3 | Day 4 | -0.123345 | -0.264241 | 0.008928 |
| N | Day 3 | Day 5 | -0.088396 | -0.215425 | 0.052869 |
| **N *** | **Day 3** | **Day 6A** | **-0.272738** | **-0.421375** | **-0.138457** |
| **N *** | **Day 3** | **Day 6B** | **-0.14204** | **-0.283497** | **-0.002211** |
| N | Day 4 | Day 5 | 0.03784 | -0.106078 | 0.178854 |
| N | Day 4 | Day 6A | -0.149443 | -0.293104 | 0.003479 |
| N | Day 4 | Day 6B | -0.018448 | -0.155635 | 0.135622 |
| **N *** | **Day 5** | **Day 6A** | **-0.187205** | **-0.32293** | **-0.038057** |
| N | Day 5 | Day 6B | -0.056903 | -0.210451 | 0.088404 |
| N | Day 6A | Day 6B | 0.132247 | -0.015311 | 0.279824 |

**Table S5.** Posterior summary of pairwise contrasts in predicted demeanor probabilities across days of the approachability test within the threatening cue condition (Day (B) − Day (A)). Estimates are posterior medians. AF = affiliative, AG = aggressive, AN = anxious, N = neutral. Lower and upper HPD give the bounds of the 95% highest posterior density interval. Rows marked with an asterisk (*) and shown in bold indicate credible differences, where the 95% HPD interval excludes zero.

| **Demeanor** | **Day (A)** | **Day (B)** | **Median** | **Lower HPD** | **Upper HPD** |
| --- | --- | --- | --- | --- | --- |
| AF | Day 1 | Day 2 | -0.097145 | -0.215677 | 0.045119 |
| AF | Day 1 | Day 3 | -0.050858 | -0.186326 | 0.080532 |
| AF | Day 1 | Day 4 | -0.021079 | -0.159471 | 0.124055 |
| **AF *** | **Day 1** | **Day 5** | **-0.163245** | **-0.284557** | **-0.036658** |
| AF | Day 1 | Day 6A | -0.038314 | -0.167033 | 0.088747 |
| **AF *** | **Day 1** | **Day 6B** | **-0.173914** | **-0.294127** | **-0.055084** |
| AF | Day 2 | Day 3 | 0.04623 | -0.085055 | 0.187841 |
| AF | Day 2 | Day 4 | 0.078 | -0.07168 | 0.219235 |
| AF | Day 2 | Day 5 | -0.062248 | -0.189355 | 0.059452 |
| AF | Day 2 | Day 6A | 0.061079 | -0.069072 | 0.201532 |
| AF | Day 2 | Day 6B | -0.075227 | -0.196689 | 0.0493 |
| AF | Day 3 | Day 4 | 0.028318 | -0.103403 | 0.18837 |
| AF | Day 3 | Day 5 | -0.11289 | -0.237711 | 0.017073 |
| AF | Day 3 | Day 6A | 0.012189 | -0.14138 | 0.146227 |
| AF | Day 3 | Day 6B | -0.124957 | -0.255738 | 0.001405 |
| **AF *** | **Day 4** | **Day 5** | **-0.138132** | **-0.27386** | **-0.005137** |
| AF | Day 4 | Day 6A | -0.018633 | -0.152564 | 0.138414 |
| **AF *** | **Day 4** | **Day 6B** | **-0.152371** | **-0.284037** | **-0.014318** |
| AF | Day 5 | Day 6A | 0.123832 | -0.002082 | 0.259097 |
| AF | Day 5 | Day 6B | -0.01184 | -0.129812 | 0.106922 |
| **AF *** | **Day 6A** | **Day 6B** | **-0.134991** | **-0.257423** | **-0.00272** |
| AG | Day 1 | Day 2 | -0.016366 | -0.078539 | 0.033408 |
| **AG *** | **Day 1** | **Day 3** | **-0.03915** | **-0.096034** | **-0.001429** |
| AG | Day 1 | Day 4 | -0.043803 | -0.096184 | 0.0014 |
| **AG *** | **Day 1** | **Day 5** | **-0.05282** | **-0.111061** | **-0.01467** |
| AG | Day 1 | Day 6A | -0.028309 | -0.083704 | 0.01608 |
| **AG *** | **Day 1** | **Day 6B** | **-0.053955** | **-0.109508** | **-0.011222** |
| AG | Day 2 | Day 3 | -0.022375 | -0.07637 | 0.016442 |
| AG | Day 2 | Day 4 | -0.026482 | -0.081244 | 0.009 |
| **AG *** | **Day 2** | **Day 5** | **-0.034492** | **-0.08846** | **-0.002912** |
| AG | Day 2 | Day 6A | -0.011994 | -0.066504 | 0.027806 |
| **AG *** | **Day 2** | **Day 6B** | **-0.035667** | **-0.091366** | **-0.005219** |
| AG | Day 3 | Day 4 | -0.004148 | -0.036733 | 0.030408 |
| AG | Day 3 | Day 5 | -0.011608 | -0.04621 | 0.012004 |
| AG | Day 3 | Day 6A | 0.010008 | -0.02812 | 0.050922 |
| AG | Day 3 | Day 6B | -0.012644 | -0.046571 | 0.011548 |
| AG | Day 4 | Day 5 | -0.007096 | -0.035998 | 0.01438 |
| AG | Day 4 | Day 6A | 0.013663 | -0.016944 | 0.057451 |
| AG | Day 4 | Day 6B | -0.008002 | -0.041188 | 0.011228 |
| AG | Day 5 | Day 6A | 0.02132 | -0.003349 | 0.065229 |
| AG | Day 5 | Day 6B | -0.00096 | -0.02306 | 0.017534 |
| AG | Day 6A | Day 6B | -0.022822 | -0.062865 | 0.00505 |
| AN | Day 1 | Day 2 | -0.000534 | -0.025414 | 0.027432 |
| **AN *** | **Day 1** | **Day 3** | **0.051485** | **0.008461** | **0.117515** |
| AN | Day 1 | Day 4 | 0.010424 | -0.017175 | 0.049784 |
| AN | Day 1 | Day 5 | -0.00818 | -0.029676 | 0.012882 |
| AN | Day 1 | Day 6A | -0.007083 | -0.031054 | 0.013051 |
| AN | Day 1 | Day 6B | 0.005717 | -0.019887 | 0.041437 |
| **AN *** | **Day 2** | **Day 3** | **0.052163** | **0.005178** | **0.11998** |
| AN | Day 2 | Day 4 | 0.01144 | -0.022446 | 0.051205 |
| AN | Day 2 | Day 5 | -0.007256 | -0.033738 | 0.018231 |
| AN | Day 2 | Day 6A | -0.005901 | -0.03484 | 0.018179 |
| AN | Day 2 | Day 6B | 0.00676 | -0.024295 | 0.045948 |
| AN | Day 3 | Day 4 | -0.040381 | -0.110258 | 0.011893 |
| **AN *** | **Day 3** | **Day 5** | **-0.060524** | **-0.123846** | **-0.012407** |
| **AN *** | **Day 3** | **Day 6A** | **-0.05853** | **-0.123383** | **-0.01044** |
| AN | Day 3 | Day 6B | -0.045046 | -0.112548 | 0.005677 |
| AN | Day 4 | Day 5 | -0.01857 | -0.057969 | 0.007429 |
| AN | Day 4 | Day 6A | -0.017916 | -0.059948 | 0.008892 |
| AN | Day 4 | Day 6B | -0.004247 | -0.047022 | 0.034237 |
| AN | Day 5 | Day 6A | 0.001189 | -0.020097 | 0.020989 |
| AN | Day 5 | Day 6B | 0.014658 | -0.01106 | 0.049787 |
| AN | Day 6A | Day 6B | 0.012829 | -0.012715 | 0.049875 |
| N | Day 1 | Day 2 | 0.113655 | -0.042994 | 0.251812 |
| N | Day 1 | Day 3 | 0.036649 | -0.105767 | 0.193922 |
| N | Day 1 | Day 4 | 0.057502 | -0.091964 | 0.212237 |
| **N *** | **Day 1** | **Day 5** | **0.228646** | **0.092615** | **0.36003** |
| N | Day 1 | Day 6A | 0.079533 | -0.06089 | 0.222246 |
| **N *** | **Day 1** | **Day 6B** | **0.22289** | **0.092377** | **0.360579** |
| N | Day 2 | Day 3 | -0.075036 | -0.235311 | 0.074031 |
| N | Day 2 | Day 4 | -0.060557 | -0.220022 | 0.086647 |
| N | Day 2 | Day 5 | 0.111121 | -0.019532 | 0.251735 |
| N | Day 2 | Day 6A | -0.038205 | -0.186175 | 0.111163 |
| N | Day 2 | Day 6B | 0.110687 | -0.038346 | 0.243098 |
| N | Day 3 | Day 4 | 0.019122 | -0.146048 | 0.167467 |
| **N *** | **Day 3** | **Day 5** | **0.189997** | **0.055974** | **0.35034** |
| N | Day 3 | Day 6A | 0.038498 | -0.108208 | 0.204583 |
| **N *** | **Day 3** | **Day 6B** | **0.187992** | **0.030872** | **0.322055** |
| **N *** | **Day 4** | **Day 5** | **0.171419** | **0.025153** | **0.309722** |
| N | Day 4 | Day 6A | 0.020894 | -0.137901 | 0.172505 |
| **N *** | **Day 4** | **Day 6B** | **0.166383** | **0.02372** | **0.316585** |
| **N *** | **Day 5** | **Day 6A** | **-0.148381** | **-0.281397** | **-0.005965** |
| N | Day 5 | Day 6B | -0.003475 | -0.14557 | 0.117692 |
| **N *** | **Day 6A** | **Day 6B** | **0.146341** | **0.009544** | **0.294632** |

**Table S6.** Posterior summary of contrasts in predicted demeanor probabilities between female and male dogs (female − male) during the approachability test, averaged across days and cue conditions. Estimates are posterior medians. AF = affiliative, AG = aggressive, AN = anxious, N = neutral. Lower and upper HPD give the bounds of the 95% highest posterior density interval. Rows marked with an asterisk (*) and shown in bold indicate credible differences, where the 95% HPD interval excludes zero.

| **Demeanor** | **Median** | **Lower HPD** | **Upper HPD** |
| --- | --- | --- | --- |
| **AF *** | **-0.117025** | **-0.197094** | **-0.025742** |
| AG | -0.008616 | -0.027157 | 0.008786 |
| AN | -0.001804 | -0.015207 | 0.009537 |
| **N *** | **0.128201** | **0.030967** | **0.218206** |

**Table S7.** Posterior summary of contrasts in predicted demeanor probabilities between cue conditions (threatening − positive) on each day of the test phase. Estimates are posterior medians. AF = affiliative, AG = aggressive, AN = anxious, N = neutral. Lower and upper HPD give the bounds of the 95% highest posterior density interval. Rows marked with an asterisk (*) and shown in bold indicate credible differences, where the 95% HPD interval excludes zero.

| **Day** | **Demeanor** | **Median** | **Lower HPD** | **Upper HPD** |
| --- | --- | --- | --- | --- |
| Day 1 | AF | -0.021811 | -0.137897 | 0.080303 |
| **Day 1** | **AG *** | **0.043252** | **0.005838** | **0.101046** |
| Day 1 | AN | 0.019208 | -0.001321 | 0.051073 |
| Day 1 | N | -0.045699 | -0.188397 | 0.080455 |
| Day 2 | AF | -0.061561 | -0.165373 | 0.044496 |
| **Day 2** | **AG *** | **0.062407** | **0.003675** | **0.146756** |
| Day 2 | AN | 0.021397 | -0.002616 | 0.061497 |
| Day 2 | N | -0.030271 | -0.16775 | 0.114325 |
| Day 3 | AF | -0.047506 | -0.183096 | 0.063089 |
| Day 3 | AG | 0.021427 | -0.002879 | 0.061751 |
| Day 3 | AN | 0.028873 | -0.002193 | 0.08087 |
| Day 3 | N | -0.008688 | -0.167337 | 0.118971 |
| Day 4 | AF | -0.069377 | -0.200251 | 0.048705 |
| Day 4 | AG | 0.006086 | -0.015564 | 0.035626 |
| Day 4 | AN | 0.011272 | -0.004629 | 0.04085 |
| Day 4 | N | 0.048327 | -0.077051 | 0.195821 |
| Day 5 | AF | -0.078013 | -0.187764 | 0.033193 |
| Day 5 | AG | 0.01438 | -0.006205 | 0.047515 |
| Day 5 | AN | 0.008496 | -0.005413 | 0.03389 |
| Day 5 | N | 0.052421 | -0.07056 | 0.181544 |

**Table S8.** Posterior summary of pairwise contrasts in predicted demeanor probabilities across days of the test phase within the positive cue condition (Day (B) − Day (A)). Estimates are posterior medians. AF = affiliative, AG = aggressive, AN = anxious, N = neutral. Lower and upper HPD give the bounds of the 95% highest posterior density interval. Rows marked with an asterisk (*) and shown in bold indicate credible differences, where the 95% HPD interval excludes zero.

| **Demeanor** | **Day (A)** | **Day (B)** | **Median** | **Lower HPD** | **Upper HPD** |
| --- | --- | --- | --- | --- | --- |
| AF | Day 1 | Day 2 | -0.071903 | -0.169691 | 0.023059 |
| AF | Day 1 | Day 3 | -0.013816 | -0.118068 | 0.089069 |
| AF | Day 1 | Day 4 | 0.011714 | -0.093761 | 0.112644 |
| AF | Day 1 | Day 5 | -0.039927 | -0.143536 | 0.060297 |
| AF | Day 2 | Day 3 | 0.05728 | -0.041426 | 0.165661 |
| AF | Day 2 | Day 4 | 0.081691 | -0.025595 | 0.193212 |
| AF | Day 2 | Day 5 | 0.031332 | -0.067648 | 0.141941 |
| AF | Day 3 | Day 4 | 0.025056 | -0.085659 | 0.14103 |
| AF | Day 3 | Day 5 | -0.0268 | -0.137162 | 0.078859 |
| AF | Day 4 | Day 5 | -0.051522 | -0.164191 | 0.056891 |
| AG | Day 1 | Day 2 | 0.004933 | -0.013791 | 0.029571 |
| AG | Day 1 | Day 3 | -0.007692 | -0.024428 | 0.003565 |
| AG | Day 1 | Day 4 | -0.004522 | -0.022416 | 0.011405 |
| AG | Day 1 | Day 5 | -0.00827 | -0.025284 | 0.004117 |
| AG | Day 2 | Day 3 | -0.013019 | -0.042965 | 0.005091 |
| AG | Day 2 | Day 4 | -0.009675 | -0.041692 | 0.010279 |
| AG | Day 2 | Day 5 | -0.013499 | -0.043275 | 0.003358 |
| AG | Day 3 | Day 4 | 0.003057 | -0.010135 | 0.021149 |
| AG | Day 3 | Day 5 | -0.00038 | -0.012792 | 0.012966 |
| AG | Day 4 | Day 5 | -0.003525 | -0.020406 | 0.010585 |
| AN | Day 1 | Day 2 | -0.001706 | -0.01205 | 0.00789 |
| AN | Day 1 | Day 3 | 0.000216 | -0.010955 | 0.012072 |
| AN | Day 1 | Day 4 | -0.002104 | -0.011605 | 0.007441 |
| AN | Day 1 | Day 5 | -0.002621 | -0.012453 | 0.006319 |
| AN | Day 2 | Day 3 | 0.001969 | -0.01088 | 0.015727 |
| AN | Day 2 | Day 4 | -0.000366 | -0.012484 | 0.009467 |
| AN | Day 2 | Day 5 | -0.000744 | -0.013284 | 0.008807 |
| AN | Day 3 | Day 4 | -0.002354 | -0.018242 | 0.008581 |
| AN | Day 3 | Day 5 | -0.002855 | -0.016893 | 0.007981 |
| AN | Day 4 | Day 5 | -0.000381 | -0.01124 | 0.00893 |
| N | Day 1 | Day 2 | 0.066535 | -0.037282 | 0.169714 |
| N | Day 1 | Day 3 | 0.022292 | -0.087943 | 0.12799 |
| N | Day 1 | Day 4 | -0.004603 | -0.11666 | 0.101594 |
| N | Day 1 | Day 5 | 0.051433 | -0.049761 | 0.16666 |
| N | Day 2 | Day 3 | -0.043984 | -0.159196 | 0.061809 |
| N | Day 2 | Day 4 | -0.069191 | -0.182486 | 0.048559 |
| N | Day 2 | Day 5 | -0.014801 | -0.126223 | 0.098856 |
| N | Day 3 | Day 4 | -0.025367 | -0.140588 | 0.093017 |
| N | Day 3 | Day 5 | 0.030756 | -0.0838 | 0.14011 |
| N | Day 4 | Day 5 | 0.056863 | -0.055568 | 0.172342 |

**Table S9.** Posterior summary of pairwise contrasts in predicted demeanor probabilities across days of the test phase within the threatening cue condition (Day (B) − Day (A)). Estimates are posterior medians. AF = affiliative, AG = aggressive, AN = anxious, N = neutral. Lower and upper HPD give the bounds of the 95% highest posterior density interval. Rows marked with an asterisk (*) and shown in bold indicate credible differences, where the 95% HPD interval excludes zero.

| **Demeanor** | **Day (A)** | **Day (B)** | **Median** | **Lower HPD** | **Upper HPD** |
| --- | --- | --- | --- | --- | --- |
| **AF *** | **Day 1** | **Day 2** | **-0.107296** | **-0.201027** | **-0.00015** |
| AF | Day 1 | Day 3 | -0.041064 | -0.154954 | 0.062177 |
| AF | Day 1 | Day 4 | -0.03659 | -0.143708 | 0.072925 |
| **AF *** | **Day 1** | **Day 5** | **-0.096441** | **-0.208424** | **-0.001594** |
| AF | Day 2 | Day 3 | 0.068341 | -0.03133 | 0.177501 |
| AF | Day 2 | Day 4 | 0.073069 | -0.027995 | 0.17569 |
| AF | Day 2 | Day 5 | 0.01233 | -0.079173 | 0.112435 |
| AF | Day 3 | Day 4 | 0.004264 | -0.109407 | 0.111854 |
| AF | Day 3 | Day 5 | -0.055746 | -0.160087 | 0.048633 |
| AF | Day 4 | Day 5 | -0.058596 | -0.161733 | 0.049019 |
| AG | Day 1 | Day 2 | 0.022833 | -0.031236 | 0.094507 |
| AG | Day 1 | Day 3 | -0.028074 | -0.084673 | 0.006782 |
| **AG *** | **Day 1** | **Day 4** | **-0.04068** | **-0.094087** | **-0.001201** |
| AG | Day 1 | Day 5 | -0.035478 | -0.095607 | 0.000835 |
| AG | Day 2 | Day 3 | -0.052498 | -0.129225 | 0.00055 |
| **AG *** | **Day 2** | **Day 4** | **-0.065732** | **-0.142294** | **-0.012899** |
| **AG *** | **Day 2** | **Day 5** | **-0.061355** | **-0.132186** | **-0.002646** |
| AG | Day 3 | Day 4 | -0.011409 | -0.050975 | 0.018222 |
| AG | Day 3 | Day 5 | -0.006468 | -0.04652 | 0.029296 |
| AG | Day 4 | Day 5 | 0.004712 | -0.026767 | 0.038075 |
| AN | Day 1 | Day 2 | -0.000289 | -0.03556 | 0.035044 |
| AN | Day 1 | Day 3 | 0.009785 | -0.022974 | 0.064199 |
| AN | Day 1 | Day 4 | -0.00958 | -0.042685 | 0.015871 |
| AN | Day 1 | Day 5 | -0.012447 | -0.045786 | 0.012258 |
| AN | Day 2 | Day 3 | 0.009709 | -0.03156 | 0.06376 |
| AN | Day 2 | Day 4 | -0.009565 | -0.051689 | 0.021154 |
| AN | Day 2 | Day 5 | -0.012561 | -0.055326 | 0.013838 |
| AN | Day 3 | Day 4 | -0.019725 | -0.070083 | 0.014629 |
| AN | Day 3 | Day 5 | -0.022077 | -0.07624 | 0.008145 |
| AN | Day 4 | Day 5 | -0.002748 | -0.033698 | 0.020555 |
| N | Day 1 | Day 2 | 0.082031 | -0.046955 | 0.211438 |
| N | Day 1 | Day 3 | 0.058034 | -0.066049 | 0.189533 |
| N | Day 1 | Day 4 | 0.092512 | -0.037107 | 0.222836 |
| **N *** | **Day 1** | **Day 5** | **0.149862** | **0.031647** | **0.274729** |
| N | Day 2 | Day 3 | -0.023354 | -0.164026 | 0.109708 |
| N | Day 2 | Day 4 | 0.007338 | -0.125353 | 0.129566 |
| N | Day 2 | Day 5 | 0.066552 | -0.060266 | 0.181772 |
| N | Day 3 | Day 4 | 0.032446 | -0.088998 | 0.169364 |
| N | Day 3 | Day 5 | 0.088571 | -0.038264 | 0.209571 |
| N | Day 4 | Day 5 | 0.058553 | -0.06658 | 0.168464 |

**Table S10.** Posterior summary of contrasts in predicted demeanor probabilities between female and male dogs (female − male) during the test phase, averaged across days and cue conditions. Estimates are posterior medians. AF = affiliative, AG = aggressive, AN = anxious, N = neutral. Lower and upper HPD give the bounds of the 95% highest posterior density interval. Rows marked with an asterisk (*) and shown in bold indicate credible differences, where the 95% HPD interval excludes zero.

| **Demeanor** | **Median** | **Lower HPD** | **Upper HPD** |
| --- | --- | --- | --- |
| AF | -0.022306 | -0.085709 | 0.036349 |
| AG | -0.000182 | -0.019373 | 0.019797 |
| AN | -0.001446 | -0.015309 | 0.011619 |
| N | 0.024448 | -0.040131 | 0.096363 |

**Data availability**

The data supporting this article are available from Mendeley Data: 10.17632/bdbg326hc7.3. Supplementary videos (Videos S1-S3) are provided as electronic supplementary material.

**Link to supplementary videos**

[Video S1](https://drive.google.com/drive/u/0/folders/1q_KVtrr0QKNHFQvlR45_UdV072ZqMN4R)

[Video S2](https://drive.google.com/drive/u/0/folders/1q_KVtrr0QKNHFQvlR45_UdV072ZqMN4R)

[Video S3](https://drive.google.com/drive/u/0/folders/1q_KVtrr0QKNHFQvlR45_UdV072ZqMN4R)

**Link to R Script file**

[RScript_file](https://drive.google.com/drive/u/0/folders/1q_KVtrr0QKNHFQvlR45_UdV072ZqMN4R)
