# Supplementary material for: Operant conditioning in Indian free-ranging dogs: effects of positive and threatening cues on sociability
Source: R Soc Open Sci. 2026 Nov 11;13(11):261465. doi: 10.1098/rsos.261465 (PMC13752531; doi:10.1098/rsos.261465)
Supplement: Supplementary file 1 [file rsos.261465.zip › rsos.261465/rsos261465_si_007.docx]

**Operant Conditioning in Indian Free-Ranging Dogs: Effects of Positive and Threatening Cues on Sociability**

Electronic Supplementary Materials

ESM file 1:

Title: ESM Figure 1 and data tables.

Legend: ESM Figure 1 shows the locations at which the experiments were performed.

ESM file 2:

Title: Video S1 - approachability test

Legend: A video showing the approachability test on Day 1

ESM file 3:

Title: Video S2 - threatening cue

Legend: A video showing the presentation of the threatening cue to a dog group

ESM file 4:

Title: Video S3 - positive cue

Legend: A video showing the presentation of the positive cue to a group of dogs

ESM file 5:

Title: Infographic of the study

Legend: An infographic summary of the study for a general reader

ESM file 6:

Title: Data

Legend: An Excel file containing the raw data of the experiment

ESM file 7:

Title: Readme file

Legend: A readme file that will enable one to understand the data file’s organization
